# Space-Time Analysis of Burgeoning US Atrial Septal Defect Rates Driven by Cannabis

**DOI:** 10.3390/jox16020068

**Published:** 2026-04-14

**Authors:** Albert Stuart Reece, Gary Kenneth Hulse

**Affiliations:** 1 Division of Psychiatry, University of Western Australia, Stirling Hwy, Crawley, Perth, WA 6009, Australia; 2 School of Medical and Health Sciences, Edith Cowan University, Joondalup Dr., Joondalup, WA 6027, Australia

**Keywords:** cannabis, cardiogenesis, genotoxicity, epigenotoxicity, transgenerational inheritance

## Abstract

Atrial septal defect (ASD) has become increasingly common in the USA and now affects 1 in 11.3 children in some places, but space–time analysis has not been applied to this emerging trend. ASD rate (ASDR) data were obtained from the National Birth Defects Prevention Network 2003–2020. Substance (cigarettes, alcohol, cannabis, analgesics, cocaine) use data were obtained from the National Survey of Drug Use and Health. Income data were obtained from the US Census. Analysis was limited to the Non-Hispanic White population by technical factors. Time-sequential univariate and bivariate maps were prepared for both covariates and outcomes and their combinations. Spatial regression of the ASDR was performed using the R package splm. A total of 7.6% of data was interpolated by linear regression. A total of 110,107 ASD cases were identified amongst 17,751,437 live births in 27 US states across 10 reporting periods. Time series maps showed that ASDR showed concordant patterns with indices of cannabis use rather than other substances. This was confirmed by multivariate spatial regression where cannabis and cannabinoids alone were found to significantly relate to ASDR, with *p* = 0.00002 for cannabidiol. Cannabis legal status similarly tracked with ASDR. Compared to states where cannabis was not legal, ASDR was more prevalent in cannabis-legal states (OR = 2.73 (2.66, 2.80); E-Value 4.90 (lower C.I. 4.76)). Twenty-seven of 34 (79.4%) E-values were >9 (high range) and 34/34 were > 1.25 (causal threshold). Data show that cannabis, including cannabis legalization, is driving the US ASD epidemic. While most high-ASDR states have high rates of cannabis use, Midwestern states where cannabis is farmed, such as Kentucky, Tennessee and Missouri, do not, suggesting other routes of exposure, potentially implicating environmental contamination. ASD is a bellwether marker for cannabinoid teratogenicity, indicating that communities should carefully control cannabinoid exposure and limit transgenerational cannabinoid genotoxicity more generally.

## 1. Introduction

Recent quinquennial reports from the Centers for Disease Control (CDC)-affiliated National Birth Defects and Prevention Network (NBDPN) show an extraordinary variation in USA atrial septal defect (ASD) secundum rates (ASDR) from 0.4/10,000 live births in Maryland to 884, 849, 802, 769, 772, and 739 /10,000 live births in various ethnic groups in Nevada and Mississippi [1]. It is noteworthy that such extraordinarily high and increasing rates of teratogenicity, involving up to almost one in eleven births, have not been explored in the broader neonatal epidemiological literature.

### 1.1. Large Population Studies

The situation is rendered more poignant because several large population-based studies have linked the recent upsurge in cannabis use across Europe and the USA with ASD [1,2,3,4,5,6,7], suggesting the two trends may be related. The first large study reviewed 316,508 live births in the Hawaii state registry 1986–2002 [3]. Prenatal cannabis use was identified in 829 cases based upon self-reports and urine toxicology screens. In five cases where cannabis was the only drug used, the rate ratio of ASDR compared to the general control group was 6.12 (95%C.I. 1.98, 14.35). In a group of 12 cases where cannabis use occurred either alone or in concert with other drugs, the rate ratio was 6.69 (3.44, 11.76).

A review of 1,015,302 births in Colorado in the 2000–2014 period found that ASDR rose significantly from 299 in 2000 to 912 in 2012 (threefold; *p* < 0.0001) when the birth rate was only rising by 3.3% annually and across a period when cannabis exposure was rising but other exposure to other drugs was stationary or declining [4,8]. Using multiple regression in linear models revealed that cannabis exposure was significant, both as a main effect and in interaction with tobacco as a predictor of major cardiovascular anomalies. In regression models quartic in time, Δ9-tetrahydrocannabinol (THC) was significant as a main effect, and cannabis-time interactions were significant in first, second, third, and fourth powers of time for major cardiovascular anomalies.

A combined geospatial and causal inferential study of birth defects in the USA in 1991–2016 found that the mean nationwide ASDR rose three-fold from 27.4 to 82.8/10,000 live births during a period when there were 73,179,872 births [2]. The main focus of this study was the period 2003–2016, a period of 57,050,944 births and for which drug use data are available from the National Survey of Drug Use and Health (NSDUH). Drug use, ethnicity and median income by state were assessed. Cannabis was found to be significantly associated with ASDR in geospatial models as a main effect and in interaction with tobacco. In inverse probability-weighted robust additive and interactive generalized regression models, cannabis and ethnic cannabis exposure showed the strongest association with ASDR. Ten E-values were documented, mostly in the range of 2–3. In all cases, the lower 95% bound of the E-values was above the threshold of causality at 1.25 [9]. On the basis of consistency with other studies, the existence of multiple biologically plausible mechanisms, strength of association, fulfillment of the Hill criteria for causality, an unequivocal temporal sequence, inverse-probability weighting and pseudo-randomization of measured predictors and E-value constraint of unmeasured confounders, this relationship was found to be causal.

A study of 2,838,963 Canadian births from 1989 to 2009 found that cardiovascular defects were elevated in high-cannabis-using territories compared to the provinces with less cannabis use in the south of the country [7,10]. Since ASD is one of the most common cardiovascular anomalies, total cardiovascular anomalies were used as a surrogate marker for ASDR in this study. In sophisticated geospatial regression models, cannabis exposure was highly significant, with *p*-values < 10^−15^ both as a main effect and in interaction with tobacco and opioids and in a three-way interaction with tobacco and opioids together.

A study of twelve cardiovascular anomalies in 14 European nations in 2010–2019 with 77,410 anomaly rates found than ASDR was highly significantly related to cannabis exposure in inverse probability-weighted panel regression after adjustment for other drug exposure and median national income (*p* < 2.2 × 10^−16^), and this high level of significance was maintained under geospatiotemporal regression [5,11]. In inverse probability-weighted panel regression models, E-values for cannabis terms ranged up to 9.01 × 10^15^, with a 95% lower bound of 2.54 × 10^13^. In geospatial models, ASDR E-values ranged up to 1.54 × 10^139^, with 1.07 × 10^21^ as the 95% lower bound. The relationship was said to be causal because of the close relationship with spatiotemporal regression, the strength of association, the fulfillment of Hill’s criteria for causality, the clear temporality, the presence of multiple plausible biological pathways, particularly from epigenomics, concordance with other large studies, the use of inverse probability weighting to control measured predictors by pseudo-randomization, and the very high E-values, which constrained unmeasured confounding within impossibly high limits.

An Australian study of 513,895 births in 2008–2015 found a prevalence ratio of 3.27 (2.63, 4.08) for ASDR in high- compared to low-cannabis-use regions [12,13]. The attributable fraction in the exposed group was 68.45% (61.69%, 75.47%). Elevated E-values suggested that uncontrolled confounding was unlikely to account for these effects, and the relationship was described as being causal.

### 1.2. Potential Implications

Cardiovascular anomalies are the largest group of congenital anomalies, among which ASD is the most common [14]. Early termination of pregnancy for anomaly (ETOPFA) is not practiced for ASD. Thus, ASD may act as a bellwether biomarker for other more serious anomalies such as chromosomal and limb anomalies for which early termination is more widely practiced and which are therefore more difficult to track longitudinally.

It should be emphasized that ASD is by one of many congenital anomalies that have recently been associated with cannabinoid exposure in both experimental and epidemiological studies [6,11,15,16,17]. Cannabinoid teratogenesis is itself a subset of the larger issue of cannabinoid genotoxicity which also includes intellectual disability, cancerogenesis including heritable cancerogenesis and cellular and organismal aging [18,19,20,21,22,23,24,25,26,27,28,29]. Therefore, the implications of the present work potentially extend far beyond fetal cardiogenesis and impact the larger consideration of cannabinoid genotoxicity, including heritable genetic and epigenomic lesions.

### 1.3. Study Objectives and Hypotheses

Given that the incidence of ASD seems to be rising across USA in general, a review of this issue is timely. This study explored several pre-specified hypotheses: (1) that cannabis is driving the US ASD rise in a manner similar to that described elsewhere; (2) that elevated cannabis use is responsible for the very high incidences reported in recent years; (3) that cannabis legalization is exacerbating this trend; and (4) that some of this effect may have been due to cannabis exposure through non-inhalational routes, potentially implicating environmental contamination. The study applies the power of space–time analysis to these questions augmented by statistical tools of formal causal inference and classical epidemiology.

## 2. Materials and Methods

### 2.1. Data

Data on ASDR by ethnicity was downloaded from 12 Periodical Reports of the NBDPN1989-1990 to 2016–2020, which is affiliated with the CDC, Atlanta, Georgia [1]. These reports were for quinquennial periods, so that he middle year was taken as the indicative year (e.g., for 2016–2020, 2018 was considered the indicative year). The states studied were Arkansas, Colorado, Delaware, Georgia, Illinois, Indiana, Iowa, Kentucky, Louisiana, Maryland, Massachusetts, Michigan, Minnesota, Mississippi, Nebraska, Nevada, New Jersey, New York, North Carolina, Oklahoma, Rhode Island, Tennessee, Texas, Utah, Virginia, West Virginia and Wisconsin. The quinquennia studied were 2003–2007, 2004–2008, 2005–2009, 2007–2011, 2008–2012, 2009–2012, 2010–2014, 2011–2015, 2012–2016 and 2016–2020. The method of birth defect ascertainment in each state was taken from annual NBDPN reports and was recorded as being “active”, “passive” or “mixed”.

Data on annual drug use exposure by US state was accessed from the National Survey of Drug Use and Health (NSDUH) conducted annually by the Substance Abuse and Mental Health Administration (SAMHSA) [30]. Data for last month cigarette (cigmon), last month alcohol use (alcmon), last month binge alcohol (bngalc), alcohol dependence (abodalc), last month cannabis (mrjmon), last year analgesic misuse (anlyr), and last year cocaine use (cocyr) was accessed for each year in 2005–2018, as 2003 was the first year the NSDUH collected data on cannabis use by state, so 2005 is the first indicative year for which analysis could be prepared. Drug use by ethnicity data at both the state and federal levels was also obtained from NSDUH. Median household income by state was obtained from the US Census. Cannabis legal status was derived from online sources [31,32]. Legal status was treated as categorial rather than ordinal data. Cannabinoid data (relating to Δ9THC, cannabigerol, cannabidiol, cannabichromene) was taken from published reports [33,34,35,36,37]. In each regression, the dependent variable was the log(ASDR). Predictor covariates were drug exposures and median state income, as indicated in each table.

### 2.2. Derived Data

Within each state, the numbers of people of each ethnicity using drugs were compared to the overall prevalence of that ethnic group in the population to derive an ethnic rate of drug use (e.g., mrjRel). This was then multiplied by the level of that drug use (e.g., mrjmon) to derive an estimate of the ethnic cannabis exposure (mrjRelmrj). This was further multiplied by the THC (tetrahydrocannabinol) content for that year at the national level to derive an estimate of average Δ9THC exposure for that ethnicity (mrjRelmrj9THC) [2,38,39,40]. Cannabis legal status was broken into four categories: illegal, medical, decriminalized and legal. The two groups, medical and decriminalized, were conflated due to small numbers in each group. Cannabis legal status was dichotomized as legal v. the rest. A semi-quantitative scale was used to plot cannabis legal status, where “Illegal” status = 0.1, “Medical” status = 0.3, “Decriminalized” = 0.6 and “Legal” = 0.98. Legal status was treated as a categorical covariate for analysis rather than an ordinal covariate. State-level cannabinoid exposure was estimated by multiplying Federal level cannabinoid concentrations by the level of cannabis used in that state.

### 2.3. Statistics

Data was processed in RStudio 2025.05.0 as GUI for R 4.5.0. Data were log transformed in the interest of normality assumptions. Such covariates included the ASD rate (ASDRt), median income, and cannabis, analgesic, and cocaine use. Data was centered for all continuous regressions in the interest of minimizing collinearity. T-tests were used to compare normally distributed data; otherwise, Wilcoxson nonparametric tests were used. Data was manipulated in the tidyverse (version 2.0.0) with “dplyr” [41]. Maps were drawn using “sf” (version 1.0-21) [42], and spatial data were manipulated with “spdep” (version 1.3-11) and “spatialreg” (version 1.3-6) [43,44] and analyzed with “splm” (version 1.6-5) [45,46]. Model reduction was achieved by the classical technique of removing the least significant term [47]. Custom color palettes were prepared for univariate plots. One map used the “viridis” color palette (version 0.6.5) [48]. Bivariate maps were prepared using the package “colorplaner” run in an R4.1.1 workspace [49]. E-Values were calculated with the package “EValue” (version 4.1.3) [50,51]. epiR (version 2.0.83) was used for the analysis of 2 × 2 tables [52].

### 2.4. Geospatiotemporal Analysis

Spatial analyses do not tolerate missing data. Up to two missing data points were tolerated for each state; 19 missing data points (251extant; total dataset N = 270) were imputed into the final dataset using within-state linear interpolation/extrapolation across time using the package “zoo” (version 1.8-14) [53] for a kriging rate of 7.6%. The appropriate error structure was determined by retaining significant error terms. Spatial weight matrices were computed using “spatialreg” (version 2.0.0) and “spdep” (version 2.0.0) [43,44]. Psi (ψ) is the parameter in spatial analysis for serial autocorrelation and was retained in all spatial models. In each case, the other major spatial parameters such as rho, lambda and phi were not significant and were therefore omitted from the final models. The major models of interest were full models containing all substance and income terms in additive and interactive models. This was true both for models, including cannabis and cannabinoids. Other models were used to establish or assess a bivariate relationship or otherwise assess the behavior of smaller groups of covariates and may be considered exploratory in nature.

### 2.5. Non-Overlapping Data

As noted above, some of the quadrennia of the data overlapped. To address the potential confusion caused by this, three non-overlapping data sets were chosen for a non-overlapping spatial analysis, namely 2003–2007, 2008–2012 and 2016–2020. These results are presented separately.

### 2.6. Survey Regression

The dependent variable was averaged over 5 years but the drug use data was available on an annual basis; this disparity was addressed by applying survey regression in the R package survey (version 4.4-2) [54]. Ten relevant periods of analysis were defined. Drug use in each state in each period was averaged. The state was incorporated into the design specification of the survey regression. All survey regressions were inverse probability-weighted using R package ipw (version 1.2.1) [55].

### 2.7. Temporal Lagging

In order to address the delayed onset of exposure, temporal lagging was also conducted. Temporal lags were introduced into the independent covariates using spatialreg and spatially regressed against the ASDR.

### 2.8. E-Values

E-values were derived from survey regression models without reliance on likelihood-based inference. Point estimates and confidence intervals were obtained from survey-weighted models using Taylor linearization for variance estimation. E-values were calculated using exponentiated coefficients and their confidence limits, as recommended for ratio-scale effect measures. Because the E-value framework does not require residual variance or likelihood-based quantities, the absence of such measures in design-based models does not preclude valid E-value computation. For estimates below the null, the corresponding inverse rate ratio and inverted confidence interval were used to compute E-values on the ≥1 scale.

### 2.9. Multicollinearity Diagnostics

Conventional variance inflation factors (VIFs) are defined for ordinary least squares regression and rely on assumptions of independent and identically distributed errors that do not hold in spatial regression models with spatially correlated disturbances. In spatial error and spatial maximum-likelihood models, the error covariance matrix is non-diagonal, which invalidates the auxiliary regressions on which standard VIF calculations are based [56,57]. Consequently, VIFs are not well-defined for assessing multicollinearity in spatial models. To evaluate collinearity among covariates independently of the spatial error structure, we employed Belsley–Kuh–Welsch collinearity diagnostics based on the eigenstructure of the standardized design matrix. This approach examines condition indices and variance-decomposition proportions to identify near-linear dependencies among regressors without relying on assumptions about the error process. *p* < 0.05 was considered significant.

### 2.10. Ethics

This study was given ethical clearance from the University of Western Australia Human Research Ethics committee, number RA/4/20/4724, on 24 September 2021.

### 2.11. Data Availability

Data including computational code is publicly available through the Mendeley data repository at doi 10.17632/4xp62s957c.5.

## 3. Results

Datasets for both ASD and drug exposure were available for the period 2003–2020 (indicative years 2005–2018), so this was the period for analysis. However, ASDR data was notably incomplete, particularly amongst ethnic minorities. For this reason, consideration was limited to the Non-Hispanic white ethnicity. The initial ASDR dataset amongst Americans of European background comprised data from 42 states over 11 different time periods. As geospatial techniques do not tolerate missing data, a decision had to be made between data retention and missing data completion. Over ten time periods, 15 states had complete data, five states had one missing datapoint, and seven states had two missing datapoints. It was decided to complete this dataset by kriging using linear regression within each state to complete missing data. In this way data for twelve states was completed with 19 results for a kriging rate of 7.6%. The size of the final dataset was thus 27 states over ten data periods in 2005–2018, for a final sample size of 270. The quinquennial span of the ten periods, including their indicative years, is shown in Supplementary Table S1. The data referred to 110,107 ASD cases amongst 17,751,437 live births identified in this period. Similar techniques have been published [2,38,39,40].

Supplementary Table S2 provides the baseline data for this analysis, with the groups stratified by legal status dichotomized as legal v. the rest. Many significant differences are noted between the two groups of states.

### 3.1. Sequential Map–Graphical Analysis

**Figure 1:**
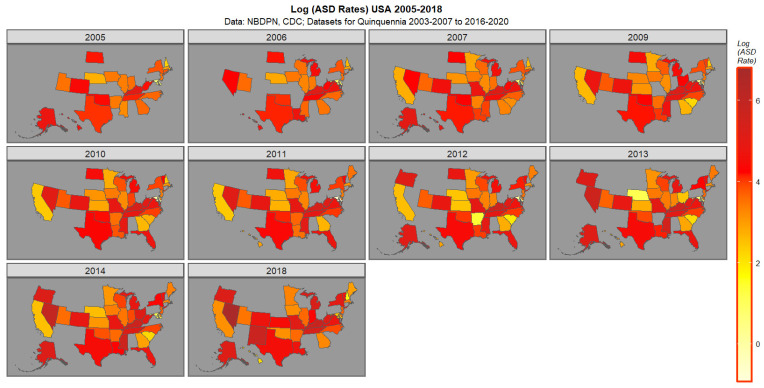
Map graph of (log) ASD Rates across USA 2005–2018.

Figure 1 presents the data for ASDR. High rates in 2018 are noted in Nevada, New Mexico, Kentucky, Tennessee, Michigan, and Missouri followed by New York, Oregon and Vermont.

Supplementary Figures S1–S6 present the spatiotemporal data for cigarettes, alcohol consumption, binge alcohol use, alcoholism, analgesic misuse, and cocaine abuse. Overall, these plots show either a decline with time or stationarity. The cocaine plot shows a recent uptick in some states in the 2016–2020 quinquennium.

**Figure 2:**
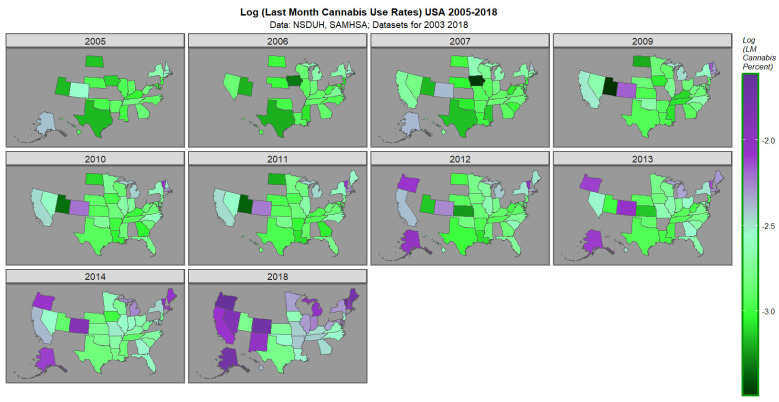
Map graph of last month cannabis use across USA 2005–2018.

The space–time plot for last cannabis use is shown in Figure 2. High rates are noted in Oregon, Nevada, Colorado, California, New Mexico, Alaska, Michigan, Main, Vermont, Massachusetts and New Hampshire.

Supplementary Figures S7–S10 present the bivariate spatiotemporal data for the ASDR against cigarettes, alcohol use disorder, analgesic misuse and cocaine abuse. Reference to the colorplane key reveals that the colors pink and purple indicate that both covariates are elevated together. Green shows where both covariates are low. Other statuses are as indicated in the key. These four plots reflect the largely disjointed time course of both covariates and the declining rate of use of the substance.

**Figure 3:**
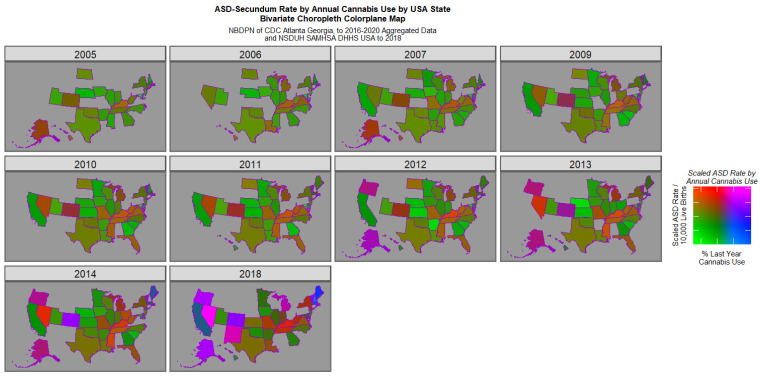
Bivariate map graph of (log) ASD rates across USA as a function of last month cannabis use. Pink and purple shades indicate where both covariates are high. Green shades indicate states were both are low. Other shades are as indicated on the colorplane key.

Figure 3 reveals the emergence of positive purple or pink signals in Colorado in 2014 and in Nevada, Oregon, Alaska, Colorado, New Mexico and Vermont in 2018. However this graph is generally suppressed by the very elevated signals in Nevada in 2014 and 2018.

**Figure 4:**
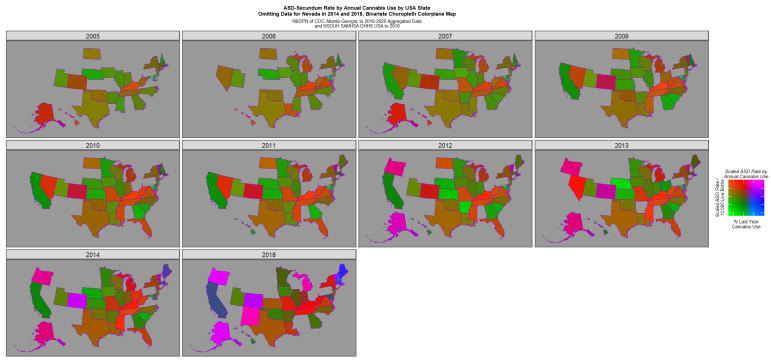
Bivariate map graph of (log) ASD rates across USA as a function of last month cannabis use (as for Figure 3) but with high rates in Nevada in 2014 and 2018 omitted and other values re-scaled appropriately to reveal the underlying trends in the next tier of states down.

Figure 4 re-plots this data with the Nevada data excised and the remaining data re-scaled appropriately. This graph reveals a bipartite picture with positive pink or purple signals coming from Oregon, Colorado, Alaska, New Mexico, Michigan and Vermont but high rates of ASD only in the Midwest states of Kentucky, Tennessee, Mississippi and Missouri.

Supplementary Figure S11 plots the cannabis legal status. Distinct similarities to Figure 2 are noted.

**Figure 5:**
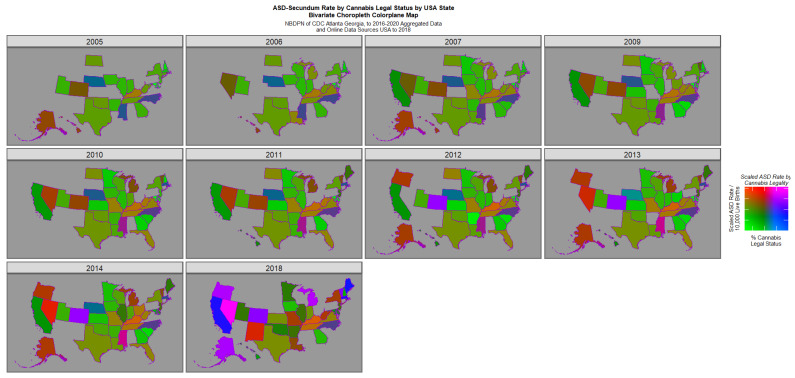
Bivariate map graph of (log) ASD rates across USA as a function of cannabis legal status with semi-quantitative scale applied to cannabis legal status, as described in Section 2.

Figure 5 is a semi-quantitative bivariate plot of cannabis legal status plotted against the ASD rate. This plot has obvious similarity to Figure 4, given the well-described elevated rate of cannabis use under more liberal legal paradigms [58,59].

### 3.2. Spatial Regression

Interstate geospatial links were computed to generate a spatial weight matrix for spatiotemporal regression (Supplementary Figure S12).

Both last month cannabis and ethnic cannabis exposure were significantly related to ASDR when considered alone in spatial regression (Table 1, first two models). When ethnic exposure to all the drugs and median income were considered, only cannabis remained in the final models (models 3 and 4). When cannabinoids were considered, both Δ9THC and cannabidiol had positive regression coefficients (model 5).

Introduction of interactions in these models produced the following results. When a tobacco: cannabis interaction was used, only the term for ethnic cannabis exposure remained positive (model 6). Then, when an interaction between Δ9THC: cannabidiol: cannabigerol was considered, terms for cannabidiol, cannabigerol and the cannabidiol: cannabigerol interaction were positive (model 7). When a tobacco interaction was added to this three-way cannabinoid interaction, no change was induced in the final model (model 8).

### 3.3. Legal Status

The levels of ASDR by legal status for the groups Illegal, Decriminalized, Medical and Legal are, respectively, 59.42 (mean, 95%C.I.; 51.71, 67.12), 55.81 (36.53, 75.10), 74.53 (54.50, 94.56), 207.00 (11.59, 402.41). These values are significantly different (ChiSqu. = 774.71, df = 711.0, *p* = 0.048).

When legal status is dichotomized as legal v. the remainder, the ASDR values for not legal and legal are 61.70 (54.78, 68.62) and 207.00 (11.59, 402.41), respectively (t = 1.45, df = 6.01, *p* = 0.19). When these data are considered as a two-by-two table, the R.R. is 2.73 (2.66, 2.80), attributable fraction in the exposed (AFE) is 3.57% (3.43%, 3.71%), Attributable Fraction in the Population (AFP) 3.55% (3.41%, 3.69%) and *p* = 0.0000. The E-value estimate is 4.90, and 4.76 is the lower bound of the 95% confidence interval.

When cannabis legal status is considered as a factor in a space–time regression, the results shown in Table 2 are derived, and “Legal” cannabis status is shown to be highly significant (model 1; β-est. = 0.47 (0.18, 0.75) *p* = 0.0013). The applicable E-values are 10.45 with 3.31 as the 95% lower bound

When dichotomized cannabis legal status is considered “Legal” cannabis status is again shown to be highly significant (β-est. = 0.34 (0.10, 0.58) *p* = 0.0059; model 2, Table 2). The applicable E-values are 6.47 with 2.23 as the 95% lower bound (with RR 3.51, (1.44, 8.54)).

When last month cannabis use was considered in an interactive spatial model along with cannabis legal status, the effect of cannabis legal status persisted in interactions (Table 2).

### 3.4. Multicollinearity Diagnostics

Belsley condition indices below conventional thresholds (10) and the absence of multiple predictors loading heavily on the same high-index dimension indicated that multicollinearity was not problematic in the geospatial models analyzed. Supplementary Table S4 presents the detailed results by covariate for each of the eight final models presented in Table 1. The Belsley collinearity diagnostics indicate no serious multicollinearity among the predictors as all condition indices are below 10, which is well under commonly accepted thresholds for concern. Variance decomposition proportions show that although the largest condition index (9.78) is associated with a relatively high proportion for CBG, no other variables share large variance decomposition proportions in that dimension, meaning no group of predictors is jointly contributing to instability. Therefore, the cannabinoid variables appear sufficiently independent for reliable regression estimation.

### 3.5. Robustness Analysis

The impact of the highest scoring state, Nevada, on these analyses was considered in a robustness analysis by analyzing the spatiotemporal dataset without Nevada. The results of space–time regression are shown in Supplementary Table S5, and the applicable E-values and relative risks are shown in Supplementary Table S6. Results of this analysis essentially confirms the general analysis shown in Table 1.

The ASD Rates for the 19 state–year combinations was also imputed by the last-observation-carried-forward technique. This technique confirmed the analysis and conclusions presented above.

### 3.6. Survey Regression

This dataset had an unusual structure since the ASDR’s were provided in quinquennial segments but the drug exposure data was available on an annual basis (Supplementary Table S1). This was addressed by the use of survey regression, with drug use data averaged within each quinquennium and the periods of analysis incorporated into the regression structure of the survey models. As shown in Table 3, these additive and interactive analyses were strongly confirmatory of the geospatial analysis but at much higher levels of statistical significance. The relative risks and E-values for all covariates from these regression summaries are shown in Supplementary Table S3.

### 3.7. Non-Overlapping Dataset

One issue with the above analysis is that some of the data is for overlapping time periods. Therefore, a new analysis was performed in which used the data from the 2003–2007, 2008–2012 and 2016–2020 periods were used. As shown in Supplementary Table S7 for additive and interactive models of both cannabis and cannabinoids, cannabis and cannabinoids were strongly associated with ASDR in all models.

### 3.8. Temporal Lagging

Another potential issue is the use of data that was not temporally lagged. Since human gestation normally takes about nine months, it may be argued that a more realistic manner in which to proceed would be to consider lagging the predictor covariates by one year to better model the biology of the exposure–outcome paradigm. The results of this analysis are presented in Supplementary Table S8 for additive and interactive models of both cannabis and cannabinoids. In each case, cannabis and cannabinoids are strongly associated with ASDR.

### 3.9. Case Ascertainment

It may also be hypothesized that the method of case ascertainment within each registry might contribute to the ASDR reported in that state. For this reason, case ascertainment styles were extracted from the annual NBDPN reports and tabulated against the ASDR. The results of this analysis are shown in Supplementary Figure S13 as both groups over aggregated time and jittered scatterplots across time. There were 93, 103 and 74 registries in the passive, mixed and active groups, respectively. The median (IQR) rates in the passive, mixed and active case ascertainment registries were 73.9 (26.7, 113.0); 36.2 (24.5, 68.2) and 34.9 (26.9, 56.5), and these were not found to be statistically different (ChiSqu. = 503, df = 464, *p* = 0.10). Thus, contrary to what might otherwise be expected, case ascertainment did not play an obvious role in systematically contributing to ASDR.

### 3.10. Sensitivity Analysis

E-values were calculated to assess the minimum strength of unmeasured confounding and their relationships with both the predictor and outcome covariates to explain away the observed associations, conditional on the ecological and design-based nature of the analysis. E-Values from the above regression and bivariate comparisons are summarized (Table 4). In total, 35/44 (79.5%) E-value estimates are seen to exceed 9, and all surpass the threshold of causality at 1.25; 12/44 (27.3%) lower bounds exceed 9, and all exceed 1.25. The median (IQR) of the E-values is 13.78 (9.25, 123.62), and the median (IQR) of the 95% lower confidence interval of the E-values is 3.31 (1.48, 9.53). These data demonstrate that results are robust to unmeasured confounding.

## 4. Discussion

### 4.1. Main Results

Study findings indicate an association of the ASDR and cannabis use. Indeed, in multivariate spatial models, cannabis use was the only covariate to survive model reduction. Plotting cannabis legal status across space and time phenocopied last month cannabis use, likely through the well-described effect of cannabis legalization increasing cannabis use [58,59]. This finding is concordant with earlier studies in locations including Hawaii, Colorado, Canada, Australia, the USA and Europe [2,3,4,5,6,7]. Of note, the strength of the association reported in this study was higher than in earlier reports, as revealed by the median E-values. Data were further supported and reinforced by the demonstration of closely concordant results using non-overlapping and temporally lagged spatial analyses and by analyses which omitted Nevada, the US State with the highest ASDRs. Taken together, the convergence of results across IPW-weighted survey models and spatial regression approaches, along with large E-values and associated analyses, provides convergent evidence consistent with a causal interpretation, although residual confounding cannot be completely excluded.

One important exception to this overall pattern occurred in the Midwestern states of Kentucky, Tennessee, Mississippi, and Missouri, where elevated rates of ASD were not accompanied by high rates of cannabis use in NSUDH. These States are known to grow large cannabis crops [60,61]. One speculative possibility is that cannabinoid exposure may occur through environmental contamination as an inevitable consequence of large-scale cannabis cultivation where plant refuse and debris contaminate rivers, streams and lakes and thereby the local water supply and food chain [62]. This has been documented around the Great Lakes and west coast waterways [63,64,65,66]. Demonstration of such a possibility would require further study. An alternative possibility is the existence of busy local cannabis dispensaries [67].

One of remarkable study feature is the very elevated ASDRs reported. Teratology rates of almost one in eleven are otherwise unusual in the neonatal literature. This is, however, consistent with the well-described exponential dose–response relationship seen in numerous laboratory studies of cannabinoid genotoxicity [68,69,70]. The implications of this exponential relationship for public health are far-reaching since it implies that relatively modest increases in teratogenic exposure can induce disproportionate rises in teratological outcomes. This issue of the supra-exponential rise in the ASDR has been considered formally [71]. That work also found a four-fold rise in the ASDR across the period 2003–2020.

The geospatial techniques used in this study do not allow temporal lags to be introduced. However since cardiogenesis occurs within the first three months of gestation, this technique should suffice to cover this biogenic period.

The method of state congenital anomaly registry case ascertainment did not appear to contribute systematically to ASDRs. Omitting the highest scoring state, Nevada, from the analysis did not materially impact the main conclusions.

This study reported higher ASDRs in states where cannabis was legal than elsewhere. This theme is considered in more detail in a companion paper [71].

### 4.2. Mechanisms

Central to any consideration of the link between defined exposures and specific outcomes is the existence of biologically plausible mechanisms that are critical to any potential causal mechanistic pathway. For this reason, a brief consideration of cardiac developmental embryology and cardiogenic epigenomics is relevant to this discussion. It is noted that this is external evidence sourced from independent investigators and does not arise from the present investigation.

During embryological development, the heart and great vessels are known to form from a complex coalescence of cells from several cardiogenic fields including the primary, secondary, and lateral heart fields, the proepicardium, the nuchal crest, and from the somites of the pharyngeal arches [72]. Atrial septal formation occurs when an initial atrial septum primum forms, degenerates later, and is superseded by the atrial septum secundum, involving complex cellular dynamics [72].

Cardiac development occurs through the sequential activation of cardiogenic gene cassettes, which are controlled by gradients of several tissue morphogens governing the development of the central cardiovasculature, along with epigenomic activation of relevant genes, both of which are widely disrupted by cannabinoids [72,73,74,75,76].

Key genes involved in cardiogenesis including sonic hedgehog (shh), VEGF, notch, Eph and Ephrin [72] have all been shown to be disrupted epigenomically by cannabis [74]. The delicate cellular choreography of cardiogenesis is controlled by gene expression and the concentration of local morphogen gradients which guide and direct cell migration and development. Sonic hedgehog (shh) is a key morphogen which is known to play a critical role in the development of the heart and other body tissues. Shh is known to be blocked directly by both Δ9THC and cannabidiol, amongst other cannabinoids [77], and epigenetically by cannabis [74]. Similarly, many other key cardiogenic morphogens [78] are inhibited both directly and epigenetically by cannabis, including fibroblast growth factor (FGF), bone morphogenetic proteins (BMP), retinoids, notch, Wnt and hippo signaling [5,17,74,75,79,80,81,82,83,84].

For example BMPs are involved in heart tube formation, formation of the cardiac outflow tract, and endocardial cushion and heart valve formation [78,85,86]. A double feedforward mechanism involving BMP4/Smad1/5, Wnt3/Tcf3 and Nodal/Smad2/3 has been described in which they cooperate to open mesoderm enhancers, affecting the expression of cardiac lineage genes [78]. Indeed, morphogens such as BMP, notch and TGF-β operate collaboratively to orchestrate cardiac outflow tract and valvular development [85].

FGF8, FGF9, FGF10, and FGF16 are involved in embryonic heart development and act as paracrine signals, influencing processes such as cardiac progenitor cell proliferation, differentiation, and patterning [87]. FGF8 is essential for the migration and differentiation of cardiac progenitor cells within the anterior heart field, which contributes to the formation of the right ventricle and outflow tract [88]. FGF10 expression is regulated by key transcription factors like ISL1, NKX2-5, and TBX1 and is crucial for the deployment of cardiogenic progenitors from the secondary heart field [89].

### 4.3. Causality

The Hill criteria are frequently used to bridge association to causation. Data in the present study and others establish that cannabis fulfills all of these criteria of strength of association, consistency amongst other series, temporality, external coherence, biological pathways, dose–response effects, other locations and experimentation. Key amongst these are cellular mechanisms that might give rise to the observed effects. As discussed briefly above and elsewhere, there are many mechanisms linking cannabis exposure to defective cardiogenesis [5,17,20,74,77,90].

Beyond the high E-values and low *p*-values reported herein, several technical features of this analysis point to a strong association between cannabis and the ASDR. Th study’s findings that cannabis was the most robust predictive covariate when both were considered covarying across space and time points to a close association. The survey regressions were inverse probability-weighted, which established a pseudo-randomized analytical paradigm in this dataset. The use of E-Values tightly constrained the potent effect demanded of any unmeasured confounding covariate. The median E-value was 13.78, with a median lower bound of 3.31. This establishes the strength of the association some unmeasured confounder must have with both the measured covariates and the outcome variable to explain the close relationship observed. It should also be noted that while E-values can be informative, they do not resolve ecological fallacy, measurement error, selection bias, or outcome misclassification problems.

It should also be noted that several interaction terms produced extremely large or infinite risk ratio estimates, resulting in correspondingly infinite E-values. Such estimates reflect sparse data or near-separation in higher-order interaction terms rather than meaningful causal effects. Thus very elevated E-values have limited practical meaning due to confounding in the context of an ecological study.

Having made these observations, the present study is strictly ecological in design. No individual-level participant data was available to the researchers. This design prevents the drawing of causal inferences in an absolute sense. Results are interpreted as population effects under measured covariate control under the standard assumptions of consistency, positivity, conditional exchangeability and correct model specification. The issue of causality is explicated in detail elsewhere [2,17,71,91]. This study can therefore be regarded as demonstrating a close association and is thus primarily hypothesis-generating.

### 4.4. Generalizability

Many features of the present study suggest that the main results are widely generalizable. Confirmatory results were obtained with bivariate and multivariate geospatial regression techniques, Chi squared tests and t-tests of inter-group comparisons and E-values. Thus, the analysis was very internally consistent. Elevated E-values indicate the result is robust to external confounding. External validation is confirmed by previous large studies from many sites that all draw similar conclusions [2,3,4,5,6,7].

### 4.5. Strengths and Limitations

This study has many strengths, including the use of the NBDPN ASD dataset, the use of space–time analyses, the confirmation of the geospatial analysis results by survey regressions, and the absence of multiple collinearity and E-values, which are one of the principle statistical tools of causal inference. Robustness, sensitivity, temporal lagged, inverse probability weighted survey regression, and non-overlapping spatiotemporal models were also examined and produced uniformly concordant results. Study weaknesses include limitation to only a single race due to the technical limitations of space–time regression, as high missing data rates limited the application of space–time methods to other ethnicities. The lack of individual-level cannabis and ASD data is a limitation shared by many other epidemiological studies. State-level associations do not necessarily reflect individual-level causal effects, and aggregate confounding (e.g., states with liberal cannabis policy may differ systematically in healthcare access, screening intensity, or reporting) introduces possible errors which cannot be ruled out regardless of E-value magnitude. Other ethnicities are addressed in a companion paper [91]. Earlier single-center retrospective studies have questioned CDC ASDR data integrity based on the finding of 64.3% accuracy in a single-center pediatric population [92]. As shown in Table 3, for that reference, the accuracy of ASD diagnosis was 19% in patients aged 41–64 years, 23% in age group 21–40 years and 64% < 21 years. This trend suggests increasing rates of diagnostic accuracy in the younger cohorts considered, and it may be reasonably expected that in the very young cohorts reported by the NBDPN, younger than 5 years old, this accuracy is significantly higher again. Whilst this issue relates to this work, it is unlikely to invalidate its main conclusions. This was an ecological study, which limits its ability to formally establish causal relationships. Some covariates were not measured in the present work, including maternal age, obesity, pregestational diabetes, exposure to teratogenic medications (antiepileptics, lithium), folic acid supplementation, environmental pollution, and access to prenatal care. These would be issues which could be taken up in subsequent research. It should also be noted that the NSDUH reflects the general population, not pregnant individuals, and state-level cannabis metrics may be proxies for multiple correlated factors (policy environment, healthcare access, socioeconomic patterns). Having said that, it has been shown several times that cannabis use by pregnant mothers closely parallels that of the general community [93,94,95,96]. Other covariates which may account for some of the marked variance in ASDRs such as changes in prenatal/postnatal imaging, screening intensity, and reporting completeness over time were not available to the present analysis but may be examined in future research. However it seems unlikely to us that such putative case ascertainment practices would co-vary across space and time coincident and concurrent with spatiotemporal cannabis distribution.

## 5. Conclusions

Many important conclusions stem from this study. The data demonstrate a close relationship between population cannabis and cannabinoid exposure with the current rising US ASD trend which, in some places, is rising to extremely high levels, approaching almost one in eleven births. Elevated cannabis-associated ASDRs may be an example of intergenerational teratogenicity and thus the transgenerational transmission of genotoxic effects. Several technical aspects of the present report support the strength of this association, including the close co-variation of cannabis exposure across space and time, the concordance between two different forms of multiple regression, the use of pseudo-randomization in the survey regressions, the low *p*-values and very high E-values, numerous cellular and molecular mechanistic pathways implicating cannabinoid genotoxicity and epigenotoxicity, obvious temporality, and concordance with many other large recent reports from other nations. Several cannabinoids may be implicated, including cannabidiol, Δ9THC, and cannabigerol, consistent with cannabinoid genotoxicity being a class effect across all cannabinoid species. Concerningly, findings in several Midwest States do not agree with local cannabis smoking trends, as documented in national surveys, but may relate to large cannabis crops grown in those vicinities, thereby potentially implicating either other exposure routes or possibly local marketing practices. This study extends earlier work by showing that the population cannabis use–ASDR association is much stronger than previously described. Overall, the findings strongly suggest that communities need to give careful consideration to the genotoxic and epigenotoxic activities of cannabinoids in designing environmentally prudent and sustainable public health policies to protect the health of current generations and the epigenetic health of generations yet to come. Further research on these important topics is strongly indicated.

## Figures and Tables

**Table 1:** Final geospatial regression models.

Parameters			Model	
Parameter	Estimate (C.I.)	*p*-Value	Metric	Value
*Model 1: Bivariate—Last Month Cannabis*			Psi	0.9690
ASD~Last Month Cannabis			Psi *p*-Value	<2.0 × 10^−16^
Last Month Cannabis	0.52 (0.30, 0.73)	2.56 × 10^−6^	LogLik.	−34.5587
			S.D.	0.2391
			AIC	75.1169
*Model 2: Bivariate—Ethnic Cannabis Exposure*			Psi	0.9692
ASD~Ethnic.Cannabis			Psi *p*-Value	<2.0 × 10^−16^
Ethnic.Cannabis	0.51 (0.30, 0.72)	2.85 × 10^−6^	LogLik.	−32.7988
			S.D.	0.2375
			AIC	71.5976
*Model 3: Additive—All Drugs*			Psi	0.9692
ASD~Ethn.Cigarettes + Ethn.Binge.Alcohol + Ethn.Cannabis + Ethn.Analgesics + Ethn.Cocaine			Psi *p*-Value	<2.0 × 10^−16^
Ethnic.Cannabis	0.51 (0.30, 0.72)	2.85 × 10^−6^	LogLik.	−32.7988
			S.D.	0.2375
			AIC	71.5976
*Model 4: Additive—All Drugs and Income*			Psi	0.9692
ASD~Ethn.Cigarettes + Ethn.Binge.Alcohol + Ethn.Cannabis + Ethn.Analgesics + Ethn.Cocaine + Income			Psi *p*-Value	<2.0 × 10^−16^
Ethnic.Cannabis	0.51 (0.30, 0.72)	2.85 × 10^−6^	LogLik.	−32.7988
			S.D.	0.2375
			AIC	71.5976
*Model 5: Additive—All Drugs with Cannabinoids*			Psi	0.9694
ASD~Ethn.Cigarettes + Ethn.Binge.Alcohol + THC + CBD + CBG + Ethn.Analgesics + Ethn.Cocaine			Psi *p*-Value	<2.0 × 10^−16^
Δ9THC	0.44 (0.08, 0.81)	0.0180	LogLik.	−35.9896
CBD	0.17 (0.09, 0.25)	2.16 × 10^−5^	S.D.	0.2402
CBG	−0.39 (−0.74, −0.04)	0.0281	AIC	81.9791
Model 6: Interactive—Cigarettes * Cannabis			Psi	0.9705
ASD~Ethn.Cigarettes * Ethn.Cannabis + Ethn.Binge.Alcohol + Ethn.Analgesics + Ethn.Cocaine + Income			Psi *p*-Value	<2.0 × 10^−16^
Ethnic.Cannabis	0.41 (0.18, 0.64)	4.07 × 10^−4^	LogLik.	−31.2932
Ethnic.Cannabis: Ethnic.Cigarettes	−1.05 (−1.86, −0.24)	0.0110	S.D.	0.2357
			AIC	70.5865
Model 7: Interactive—Cannabinoids—THC * CBD * CBG			Psi	0.9696
ASD~Ethn.Cigarettes + Ethn.Binge.Alcohol + THC * CBD * CBG + Ethn.Analgesics + Ethn.Cocaine + Income			Psi *p*-Value	<2.0 × 10^−16^
Δ9THC: CBD	1.08 (0.28, 1.89)	0.0085	LogLik.	−34.7259
CBD: CBG	0.44 (0.16, 0.72)	0.0023	S.D.	0.2390
			AIC	77.4519
Model 8: Interactive—Cannabinoids—Cigarettes * THC * CBD * CBG			Psi	0.9698
ASD~Ethn.Cigarettes * THC * CBD * CBG + Ethn.Binge.Alcohol + Ethn.Analgesics + Ethn.Cocaine + Income			Psi *p*-Value	<2.0 × 10^−16^
Δ9THC: CBD	1.08 (0.28, 1.89)	0.0085	LogLik.	−31.9786
CBD: CBG	0.44 (0.16, 0.72)	0.0023	S.D.	0.2366
			AIC	75.9573

Notes: Psi—Psi (ψ) is the parameter in spatial analysis for serial autocorrelation. LogLik.—Log likelihood ratio at model optimization. S.D.—Standard deviation. AIC—Akaike Information Criterion. *—Interaction.

**Table 2:** Geospatial regression models of cannabis legal status.

Parameters			Model	
Parameter	Estimate (C.I.)	*p*-Value	Metric	Value
*Legal Status*			Psi	0.9669
ASD~Legal Status			Psi *p*-Value	<2.0 × 10^−16^
Legal Cannabis	0.47 (0.18, 0.75)	0.0013	LogLik.	−46.2190
			S.D.	0.2505
			AIC	102.4380
*Legal Status—Dichotomized*			Psi	0.9675
ASD~Legal Status Dichotomized			Psi *p*-Value	<2.0 × 10^−16^
Legal (Dichotomized v Others)	0.34 (0.1, 0.58)	0.0059	LogLik.	−41.4375
			S.D.	0.2459
			AIC	88.8751
*Additive: Legal Status + Last.Month.Cannabis*			Psi	0.9690
ASD~Legal Status + Last.Month.Cannabis			Psi *p*-Value	<2.0 × 10^−16^
Last.Month.Cannabis	0.52 (0.3, 0.73)	2.56 × 10^−6^	LogLik.	−34.5585
			S.D.	0.2391
			AIC	75.1169
Interactive: Legal Status * Last.Monthy.Cannabis			Psi	0.9706
ASD~Legal Status * Last.Month.Cannabis			Psi *p*-Value	<2.0 × 10^−16^
StatusMedical	1.81 (0.41, 3.21)	0.0112	LogLik.	−28.9705
StatusDecriminalized: Cannabis	0.82 (0.08, 1.56)	0.0306	S.D.	0.2336
StatusMedical: Cannabis	0.97 (0.47, 1.48)	0.0002	AIC	75.9410

Note: *—Interaction.

**Table 3:** Final survey regression models.

Term	Estimate (C.I.)	*p*-Value
***CANNABIS***		
***Additive***		
*ASD~Cigarettes + Cannabis + Bng.Alc + Analgesics + Cocaine + Median.Income*		
Cigarettes	−0.3 (−0.35, −0.25)	3.79 × 10^−11^
Cannabis	0.84 (0.82, 0.85)	3.10 × 10^−34^
Cocaine	−37.03 (−37.87, −36.18)	7.32 × 10^−31^
***One Interaction***		
ASD~Cigarettes * Cannabis + Bng.Alc + Analgesics + Cocaine + Median.Income		
Cigarettes	73.44 (68.91, 77.96)	3.92 × 10^−19^
Cannabis	246.31 (230.72, 261.91)	6.66 × 10^−19^
Analgesics	20.97 (20, 21.94)	1.27 × 10^−21^
Cocaine	−40.14 (−41.74, −38.53)	7.38 × 10^−23^
Median.Income	−6.90 × 10^−5^ (−7.369 × 10^−5^, −6.44 × 10^−5^)	7.74 × 10^−18^
Cigarettes: Cannabis	−1165.66 (−1238.75, −1092.57)	5.51 × 10^−19^
***Two Interactions***		
ASD~Cigarettes * Cannabis * Bng.Alc + Analgesics + Cocaine + Median.Income		
Cigarettes	−666.11 (−823.29, −508.93)	7.78 × 10^−8^
Cannabis	−3580.26 (−4160.79, −2999.74)	2.89 × 10^−10^
Analgesics	−168.29 (−214.85, −121.73)	6.96 × 10^−7^
Cocaine	415.09 (329.78, 500.4)	1.05 × 10^−8^
Median.Income	−2.12 × 10^−4^ (−2.78 × 10^−4^, −1.46 × 10^−4^)	7.36 × 10^−6^
Cigarettes: Bing.Alc	1669.78 (1377.83, 1961.73)	9.24 × 10^−10^
Cigarettes: Cannabis	24,277.95 (20,888.63, 27,667.27)	2.75 × 10^−11^
Bng.Alc: Cannabis	11,982.18 (10,384.19, 13,580.16)	1.33 × 10^−11^
Cigarettes: Cannabis: Bng.Alc	−84,238.48 (−94,661.76, −73,815.2)	3.99 × 10^−12^
***CANNABINOIDS***		
***Additive***		
*ASD~Cigarettes + THC + CBG + CBD + Bng.Alc + Analgesics + Cocaine + Median.Income*		
Cigarettes	−31.22 (−33.69, −28.75)	1.49 × 10^−17^
Binge.Alcohol	70.93 (64.95, 76.9)	5.39 × 10^−17^
THC	0.12 (0.11, 0.13)	1.39 × 10^−18^
CBG	−1.5 (−1.64, −1.36)	3.78 × 10^−16^
Median.Income	−1.37 × 10^−4^ (−1.48 × 10^−4^, −1.26 × 10^−4^)	6.11 × 10^−17^
***Two Interactions***		
ASD~Cigarettes + THC * CBG * CBD + Bng.Alc + Analgesics + Cocaine + Median.Income		
Binge.Alcohol	7.24 (7.18, 7.3)	1.98 × 10^−33^
CBG	−164.66 (−171.8, −157.52)	1.58 × 10^−20^
CBD	−41.41 (−47.83, −34.98)	7.06 × 10^−11^
Cocaine	299.73 (280.7, 318.76)	1.41 × 10^−17^
Median.Income	−3.12 × 10^−4^ (−3.48 × 10^−4^, −2.75 × 10^−4^)	1.91 × 10^−12^
THC: CBG	6.26 (6.03, 6.5)	1.47 × 10^−21^
THC: CBD	−9.59 (−10.2, −8.98)	1.34 × 10^−17^
CBG: CBD	335.41 (309.73, 361.08)	3.85 × 10^−16^
***Three Interactions***		
ASD~Cigarettes * THC * CBG * CBD + Bng.Alc + Analgesics + Cocaine + Median.Income		
Cigarettes	−336.42 (−579.69, −93.15)	0.0100
THC	−17.08 (−24.68, −9.49)	2.36 × 10^−4^
CBG	369.53 (252.33, 486.72)	6.86 × 10^−6^
Analgesics	−336.02 (−376.87, −295.18)	2.10 × 10^−11^
Cocaine	626.18 (545.08, 707.27)	5.21 × 10^−11^
Cigarettes: THC	103.2 (60.14, 146.27)	1.29 × 10^−4^
Cigarettes: CBG	−2648.53 (−3556.44, −1740.62)	1.65 × 10^−5^
Cigarettes: CBD	396 (281.75, 510.25)	2.26 × 10^−6^
THC: CBD	−37.27 (−55.53, −19.01)	5.71 × 10^−4^
Cigarettes: THC: CBG	1264.46 (614.63, 1914.29)	8.60 × 10^−4^
THC: CBG: CBD	41.12 (18.34, 63.91)	0.0016

Note: *—Interaction.

**Table 4:** E-Values from all models.

Parameter	R.R. (95%C.I.)	E-Values (& Lower Bounds)
Last.Month.Cannabis	7.15 (3.16, 16.21)	13.78, 5.76
Ethnic.Cannabis	7.08 (3.12, 16.02)	13.63, 5.70
Ethnic.Cannabis	7.08 (3.12, 16.02)	13.63, 5.70
Ethnic.Cannabis	7.08 (3.12, 16.02)	13.63, 5.70
Δ9THC	5.31 (1.34, 21.14)	10.10, 2.01
CBD	1.92 (1.42, 2.58)	3.24, 2.19
Ethnic.Cannabis	4.89 (2.03, 11.76)	9.25, 3.48
THC: CBG	62.06 (2.89, 1334.97)	123.62, 5.22
CBG: CBD	5.28 (1.82, 15.31)	10.02, 3.04
THC: CBG	62.06 (2.89, 1334.97)	123.62, 5.22
CBG: CBD	5.28 (1.82, 15.31)	10.02, 3.04
Spatial Legal Status	5.49 (1.95, 15.46)	10.45, 3.31
Spatial Legal v Others	3.51 (1.44, 8.54)	6.47, 2.23
Last.Month.Cannabis	7.15 (3.16, 16.21)	13.78, 5.76
StatusMedical	1158.92 (5.03, 26,7205.26)	2317.35, 9.53
StatusDecriminalized: log(mrjmon)	24.35 (1.36, 436.84)	48.19, 2.05
StatusMedical: log(mrjmon)	44.25 (6.16, 317.93)	87.99, 11.79
Cannabis	2.31 (2.28, 2.34)	4.05, 3.98
Cannabis	9.37 × 10^106^ (1.58 × 10^100^, 5.55 × 10^113^)	1.87 × 10^107^, 3.17 × 10^100^
Cigarettes: Cannabis	Inf (Inf, Inf)	Inf, Inf
Bng.Alc: Cannabis	Inf (Inf, Inf)	Inf, Inf
THC	1.13 (1.12, 1.14)	1.51, 1.48
THC: CBG	525.2 (414.07, 666.16)	1049.9, 827.64
CBG: CBD	4.63 × 10^145^ (3.27 × 10^134^, 6.56 × 10^156^)	9.27 × 10^145^, 6.54 × 10^134^
CBG	3.04 × 10^160^ (3.84 × 10^109^, 2.41 × 10^211^)	Inf, 7.67 × 10^109^
Cigarettes: THC	6.63 × 10^44^ (1.31 × 10^26^, 3.36 × 10^63^)	1.33 × 10^45^, 2.61 × 10^26^
Cigarettes: CBD	9.55 × 10^171^ (2.31 × 10^122^, 3.95 × 10^221^)	Inf, 4.62 × 10^122^
Cigarettes: THC: CBG	Inf (8.5 × 10^266^, Inf)	Inf, Inf
THC: CBG: CBD	7.24 × 10^17^ (9.21 × 10^8^, 5.7 × 10^27^)	1.45 × 10^18^, 1.84 × 10^10^
Cannabis (Less Nevada)	5.00 (2.10, 11.9)	9.48, 3.62
Cannabis (Less Nevada)	3.52 (1.40, 8.80)	6.49, 2.16
CBD (Less Nevada)	1.46 (1.16, 1.84)	2.28, 1.60
THC: CBG (Less Nevada)	38.79 (1.69, 890.56)	77.08, 2.77
CBG: CBD (Less Nevada)	4.52 (1.52, 13.43)	8.51, 2.41
Cannabis (Non-Overlap)	13.01 (4.45, 38.04)	25.51, 8.37
Cannabis (Non-Overlap)	13.01 (4.45, 38.04)	25.51, 8.37
THC: CBD (Non-Overlap)	9.36 (2.75, 31.79)	18.2, 4.95
THC: CBD (Non-Overlap)	7.72 (2.18, 27.37)	14.93, 3.78
Cannabis (Lagged)	7.15 (3.16, 16.21)	13.78, 5.76
Cannabis (Lagged)	4.87 (2.02, 11.71)	9.21, 3.46
THC (Lagged)	5.31 (1.34, 21.14)	10.1, 2.01
CBD (Lagged)	1.92 (1.42, 2.58)	3.24, 2.19
THC (Lagged)	5.31 (1.34, 21.14)	10.1, 2.01
CBD (Lagged)	1.92 (1.42, 2.58)	3.24, 2.19

## Data Availability

Data including computational code is publicly available through the Mendeley data repository at doi: 10.17632/4xp62s957c.5.

## Supplementary Materials

The following supporting information can be downloaded at: https://www.mdpi.com/article/10.3390/jox16020068/s1, Supplementary Tables: Table S1: Ten Quinquennial Time Periods; Table S2: Social, Demographic and Substance Exposure Table, Stratified by Dichotomized Legal Status; Table S3: Belsley Condition Matrices for the Eight Models of Table 1; Table S4: E-Values of the survey regression models. Table S5: Spatial Regression Results Without Nevada; Table S6: E-Values of Spatial Regression Results Without Nevada; Table S7: Spatial Regression of Three Non-Overlapping Periods; Table S8: Spatial Regression of Temporally Lagged Time Series. Supplementary Figures: Figure S1: Univariate map graph of cigarette use across USA 2005–2018; Figure S2: Univariate map graph of cigarette use across USA 2005–2018; Figure S3: Univariate map graph of cigarette use across USA 2005–2018; Figure S4: Univariate map graph of cigarette use across USA 2005–2018; Figure S5: Univariate map graph of cigarette use across USA 2005–2018; Figure S6: Univariate map graph of cigarette use across USA 2005–2018; Figure S7: Bivariate map graph of Atrial septal defect rates against last month cigarette use across USA 2005–2018; Figure S8: Bivariate map graph of Atrial septal defect rates against last month cigarette use across USA 2005–2018; Figure S9: Bivariate map graph of Atrial septal defect rates against last month cigarette use across USA 2005–2018; Figure S10: Bivariate map graph of Atrial septal defect rates against last month cigarette use across USA 2005–2018; Figure S11: Univariate map graph of cannabis legal status across USA 2005–2018; Figure S12: Bivariate map graph of Atrial septal defect rates against cannabis legal status across USA 2005–2018; Figure S13: ASD rates by Case Ascertainment style in each state; (**A**) Jittered scatterplot of ASD Rate over time; (**B**) Boxplot of ASD Rate by case ascertainment type; (**C**) Jittered scatterplot of log (ASD Rate) over time; (**D**) Boxplot of Log (ASD Rate) by case ascertainment type.

## Abbreviations

The following abbreviations are used in this manuscript:

**Acronym**	**Name**
AIC	Akaike Information Criterion
alcmon	Alcohol use in the past year
anlyr	Analgesic misuse in the past year
ASD	Atrial Septal Defect, secundum type
ASDR	Atrial Septal Defect Rate
audyr	Alcohol use disorder in the past year
B IC	Bayesian Information Criterion
bngalc	Binge alcohol use in the past year
BMP	Bone Morphogenetic Proteins
CBG	Cannabigerol
C.I.	Confidence Interval
cigmon	Cigarette use in the past month
cocyr	Cocaine use in the past year
E-Value	Evidence value
Δ9THC	Δ9-tetrahydrocannabinol
FGF	Fibroblast Growth Factor
IQR	Interquartile Range
mrjmon	Cannabis use in the past month
N	Number in sample size
NBDPN	National Brith Defect Prevention Network
NHWhite	Non-Hispanic White
NSDUH	National Survey of Drug Use and Health
SAMHSA	Substance Abuse and Mental Health Services Administration
SD	Standard deviation
SEM	Standard error of the mean
SHH	Sonic Hedgehog
TGFβ	Transforming Growth Factor-β
VEGF	Vascular Endothelial Growth Factor
