# Supplementary material for: Space-Time Analysis of Burgeoning US Atrial Septal Defect Rates Driven by Cannabis
Source: J Xenobiot. 2026 Apr 14;16(2):68. doi: 10.3390/jox16020068 (PMC13117238; doi:10.3390/jox16020068)
Supplement: Supplementary file 1 [file jox-16-00068-s001.zip › jox-4182013-supplementary.pdf]

SUPPLEMENTARY TABLES

Table of Contents

Table No.	Subject
S1	Ten Quinquennial Time Periods
S2	Social, Demographic and Substance Exposure Table, Stratified by Dichotomized Legal Status
S3	Belsley Condition Matrices
S4	Survey Regression E-Values
S5	Spatial Regression Results Without Nevada
S6	E-Values of Spatial Regression Results Without Nevada
S7	Spatial Regression of Three Non-Overlapping Periods
S8	Spatial Regression of Temporally Lagged Time Series

Supplementary Table S1. Periods

Period	Indicative Year	Group
2003-2007	2005	1
2004-2008	2006	2
2005-2009	2007	3
2007-2011	2009	4
2008-2012	2010	5
2009-2012	2011	6
2010-2014	2012	7
2011-2015	2013	8
2012-2016	2014	9
2016-2020	2018	10

Supplementary Table S2.
Sociodemographic and substance Exposures by Legal Status

Parameter	Not Legal	Legal	P-Value	SMD
Sample Size (N)	263	7		
ASD Rate (median [IQR])	37.60 [26.14, 80.40]	116.80 [102.60, 152.50]	0.007	0.761
Log (ASD Rate) (median [IQR])	4.31 [3.95, 5.08]	5.45 [5.32, 5.70]	0.007	1.127
Births (mean (SD))	65007 [35682, 65830]	68487 [38715, 119002]	0.002	0.243
Numbers Atrial Septal Defect (Total)	103,769	6,214	0.001	0.226
Births (Total)	17,366,382	385,055		
Last.Month.Cigarettes (median [IQR])	0.25 [0.23, 0.28]	0.21 [0.20, 0.22]	0.003	1.317
Last.Month.Alcohol (median [IQR])	0.57 [0.48, 0.60]	0.66 [0.61, 0.66]	0.010	1.149
Alcohol Dependence (median [IQR])	0.07 [0.06, 0.08]	0.08 [0.06, 0.08]	0.475	0.222
Last.Month.Binge.Alcohol (median [IQR])	0.25 [0.22, 0.27]	0.27 [0.26, 0.29]	0.047	0.818
Last.Month.Cannabis (median [IQR])	0.06 [0.05, 0.07]	0.14 [0.13, 0.15]	<0.001	3.435
Last.Year.Analgesics (median [IQR])	0.04 [0.04, 0.05]	0.04 [0.04, 0.05]	0.411	0.407
Last.Year.Cocaine (median [IQR])	0.02 [0.01, 0.02]	0.03 [0.02, 0.03]	0.001	1.464
Δ9THC.National (median [IQR])	10.00 [8.76, 14.10]	14.88 [14.35, 14.88]	0.001	1.724
Cannabidiol.National (median [IQR])	0.28 [0.20, 0.460]	0.71 [0.18 0.71]	0.301	0.595
Cannabigerol.National (median [IQR])	0.43 [0.40, 0.46]	0.58 [0.46, 0.58]	0.001	1.534
Cannabinol.National (median [IQR])	0.43 [0.37, 0.63]	0.70 [0.64, 0.70]	0.001	1.849
Cannabichromene.National (median [IQR])	0.24 [0.24, 0.26]	0.29 [0.26, 0.29]	0.032	1.009
THCV.National (median [IQR])	0.09 [0.09, 0.10]	0.19 [0.10, 0.19]	0.011	1.172
Δ9THC * LM.Cannabis (median [IQR])	0.68 [0.51, 0.92]	2.08 [1.83, 2.27]	<0.001	3.531
Median.Household.Income (median [IQR])	50219 [43634, 56994]	60940 [57426, 66091]	0.002	1.410
ASD Rate (Ethnic) (median [IQR])	37.30 [26.05, 80.40]	116.80 [102.60, 152.50]	0.007	0.763
Log ASD Rate (Ethnic) (median [IQR])	4.31 [3.95, 5.08]	5.45 [5.32, 5.70]	0.007	1.127
ASD Rate (All Races) (median [IQR])	41.20 [26.98, 86.55]	130.90 [120.95, 170.45]	0.006	0.819
Intrastate.Ethnic.Cigarettes (median [IQR])	1.10 [1.09, 1.11]	1.12 [1.12, 1.12]	0.002	1.316
Intrastate.Ethnic.Alcoholism (median [IQR])	1.05 [1.03, 1.06]	1.09 [1.05, 1.09]	0.006	1.297
Intrastate.Ethnic.Binge.Alcohol (median [IQR])	1.09 [1.08, 1.09]	1.08 [1.08, 1.09]	0.073	0.564
Intrastate.Ethnic.Cannabis (median [IQR])	1.07 [1.06, 1.07]	1.07 [1.06, 1.07]	0.697	0.553
Intrastate.Ethnic.Analgesics (median [IQR])	1.11 [1.08, 1.11]	1.09 [1.07, 1.09]	0.028	1.150
Intrastate.Ethnic.Cocaine (median [IQR])	1.08 [1.08, 1.10]	1.10 [1.09, 1.10]	0.581	0.127
Ethnic.Cigarettes (median [IQR])	0.28 [0.26, 0.31]	0.24 [0.22, 0.25]	0.003	1.264
Ethnic.Alcoholism (median [IQR])	0.07 [0.07, 0.08]	0.08 [0.07, 0.09]	0.288	0.385
Ethnic.Binge.Alcohol (median [IQR])	0.27 [0.24, 0.29]	0.30 [0.28, 0.31]	0.054	0.791
Ethnic.Cannabis (median [IQR])	0.06 [0.06, 0.08]	0.15 [0.14, 0.16]	<0.001	3.416
Ethnic.Analgesics (median [IQR])	0.05 [0.04, 0.06]	0.05 [0.04, 0.05]	0.267	0.524
Ethnbic.Cocaine (median [IQR])	0.02 [0.02, 0.03]	0.03 [0.03, 0.03]	0.001	1.448
Ethnic.Cannabis * Δ9THC (median [IQR])	0.74 [0.54, 0.98]	2.22 [1.95, 2.42]	<0.001	3.518
Status (N)			<0.001	0.960
Illegal (mean, SD)	180 (68.4)	0 (0.0)		
Medical (mean, SD)	48 (18.3)	0 (0.0)		
Decriminalized (mean, SD)	35 (13.3)	0 (0.0)		

Legal (mean, SD)	0 (0.0)	7 (100.0)		

Note:

SMD – standardized mean difference.
IQR – Interquartile range.

Supplementary Table S3. Survey Regression Model E-Values

Term	R.R. (C.I.)	E-Values
CANNABIS		
Additive		
ASD ~ Cigarettes + Cannabis + Bng.Alc + Analgesics + Cocaine + Median.Income		
Cigarettes	0.74 (0.7, 0.78)	0.49, 0.53
Cannabis	2.31 (2.28, 2.34)	4.05, 3.98
Cocaine	8.31E-17 (3.57E-17, 1.94E-16)	4.16E-17, 9.70E-17
One Interaction		
*ASD ~ Cigarettes * Cannabis + Bng.Alc + Analgesics + Cocaine + Median.Income*		
Cigarettes	7.81E+31 (8.46E+29, 7.21E+33)	1.56E+32, 1.69E+30
Cannabis	9.37E+106 (1.58E+100, 5.55E+113)	1.87E+107, 3.17E+100
Analgesics	1.29E+09 (4.08E+08, 3.36E+09)	2.56E+09, 9.75E+08
Cocaine	3.7E-18 (7.46E-19, 1.84E-17)	1.85E-18, 9.2E-18
Median.Income	1 (1, 1)	1.01, 1.01
Cigarettes: Cannabis	0 (0, 0)	0, 0
Two Interactions		
*ASD ~ Cigarettes * Cannabis * Bng.Alc + Analgesics + Cocaine + Median.Income*		
Cigarettes	5.16E-290 (0, 9.46E-222)	0, 0
Cannabis	0 (0, 0)	0, 0
Analgesics	8.16E-74 (4.92E-94, 1.35E-53)	4.08E-74, 6.77E-54
Cocaine	1.87E+180 (1.67E+143, 2.09E+217)	Inf, 3.34E+143
Median.Income	1 (1, 1)	0.99, 0.99
Cigarettes: Bing.Alc	Inf (Inf, Inf)	Inf, Inf
Cigarettes: Cannabis	Inf (Inf, Inf)	Inf, Inf
Bng.Alc: Cannabis	Inf (Inf, Inf)	Inf, Inf
Cigarettes: Cannabis: Bng.Alc	0 (0, 0)	0, 0
CANNABINOIDS		
Additive		
ASD ~ Cigarettes + THC + CBG + CBD + Bng.Alc + Analgesics + Cocaine + Median.Income		
Cigarettes	2.75E-14 (2.33E-15, 3.25E-13)	1.38E-14, 1.63E-13
Binge.Alcohol	6.36E+30 (1.61E+28, 2.5E+33)	1.27E+31, 3.23E+28
THC	1.13 (1.12, 1.14)	1.51, 1.48
CBG	0.22 (0.19, 0.26)	0.12, 0.14
Median.Income	1 (1, 1)	0.99, 0.99
Two Interactions		
*ASD ~ Cigarettes + THC * CBG * CBD + Bng.Alc + Analgesics + Cocaine + Median.Income*		
Binge.Alcohol	1390.7 (1309.71, 1476.71)	2780, 2620
CBG	3.08E-72 (2.45E-75, 3.87E-69)	1.54E-72, 1.94E-69
CBD	1.04E-18 (1.69E-21, 6.42E-16)	5.2E-19, 3.21E-16
Cocaine	1.48E+130 (8.06E+121, 2.72E+138)	2.96E+130, 1.61E+122
Median.Income	1 (1, 1)	1.02, 1.02

THC: CBG	525.2 (414.07, 666.16)	1049.9, 827.64
THC: CBD	6.85E-05 (3.73E-05, 1.26E-04)	3.43E-05, 6.29E-05
CBG: CBD	4.63E+145 (3.27E+134, 6.56E+156)	9.27E+145, 6.54E+134
Three Interactions		
*ASD ~ Cigarettes * THC * CBG * CBD + Bng.Alc + Analgesics + Cocaine + Median.Income*		
Cigarettes	7.86E-147 (1.75E-252, 3.52E-41)	3.93E-147, 1.76E-41
THC	3.81E-08 (1.92E-11, 7.58E-05)	1.91E-08, 3.79E-05
CBG	3.04E+160 (3.84E+109, 2.41E+211)	Inf, 7.67E+109
Analgesics	1.17E-146 (2.13E-164, 6.36E-129)	5.83E-147, 3.18E-129
Cocaine	8.81E+271 (5.32E+236, 1.46E+307)	Inf, Inf
Cigarettes: THC	6.63E+44 (1.31E+26, 3.36E+63)	1.33E+45, 2.61E+26
Cigarettes: CBG	0 (0, 0)	0, 0
Cigarettes: CBD	9.55E+171 (2.31E+122, 3.95E+221)	Inf, 4.62E+122
THC: CBD	6.52E-17 (7.66E-25, 5.55E-09)	3.26E-17, 2.78E-09
Cigarettes: THC: CBG	Inf (8.5E+266, Inf)	Inf, Inf
THC: CBG: CBD	7.24E+17 (9.21E+08, 5.7E+27)	1.45E+18, 1.84E+10

Supplementary Table S4. Belsley Condition Matrices for the Eight Models of Table 1

Model	Variables				Condition Index
Bivariate - Last Month Cannabis					
	Last Month Cannabis				
	1.0000				1.0000
Bivariate - Ethnic Cannabis Exposure					
	Ethnic.Cannabis				
	1.0000				1.0000
Additive - All Drugs					
	Ethnic.Cannabis				
	1.0000				1.0000
Additive - All Drugs and Income					
	Ethnic.Cannabis				
	1.0000				1.0000
Additive - All Drugs with Cannabinoids					
	Δ9THC	CBD	CBG		
	0.5208	0.0010	0.4782		1.0000
	0.1402	0.6757	0.1841		1.3073
	0.3390	0.3232	0.3377		4.5549
Interactive - Cigarettes * Cannabis					
	Ethn.Cannabis	Ethn.Cannabis: Ethn.Cigarettes			
	0.5000	0.5000			1.0000
	0.5000	0.5000			1.7383
Interactive - Cannabinoids - THC * CBD * CBG					
	Δ9THC	Δ9THC : CBD	CBD : CBG	Δ9THC : CBD : CBG	
	0.0367	0.9029	0.0447	0.0158	1.0000
	0.2723	0.0534	0.5720	0.1023	1.6137
	0.3236	0.0085	0.3592	0.3088	2.4248
	0.3675	0.0353	0.0242	0.5731	9.7758
Interactive - Cannabinoids - Cigarettes *THC * CBD * CBG					
	Δ9THC	Δ9THC : CBD	CBD : CBG	Δ9THC : CBD : CBG	
	0.0367	0.9029	0.0447	0.0158	1.0000
	0.2723	0.0534	0.5720	0.1023	1.6137
	0.3236	0.0085	0.3592	0.3088	2.4248
	0.3675	0.0353	0.0242	0.5731	9.7758

Supplementary Table S5. Spatiotemporal regressions of Dataset Omitting Nevada

Parameters			Model	
**Parameter**	**Estimate (C.I.)**	**P-Value**	**Metric**	**Value**
CANNABIS			Psi	0.9670
Additive			Psi P-Value	<2.0E-16
ASD ~ Cigarettes + Cannabis + Bng.Alc + Analgesics + Cocaine + Median.Income			LogLik.	-29.7707
Cannabis	0.42 (0.19, 0.64)	0.0003	S.D.	0.2366
			AIC	65.5414
Interactive			Psi	0.9685
*ASD ~ Cigarettes * Cannabis + Bng.Alc + Analgesics + Cocaine + Median.Income*			Psi P-Value	<2.0E-16
Cannabis	0.32 (0.09, 0.56)	0.0073	LogLik.	-26.7904
Cigarettes: Cannabis	-1.01 (-1.82, -0.21)	0.0138	S.D.	0.2334
			AIC	61.5809
CANNABINOIDS			Psi	0.9673
Additive			Psi P-Value	<2.0E-16
ASD ~ Cigarettes + THC + CBG + CBD + Bng.Alc + Analgesics + Cocaine + Median.Income			LogLik.	-31.0458
CBD	0.1 (0.04, 0.16)	0.0012	S.D.	0.2377
			AIC	68.0915
Interactive x 3			Psi	0.9674
*ASD ~ Cigarettes * THC * CBG * CBD + Bng.Alc + Analgesics + Cocaine + Median.Income*			Psi P-Value	<2.0E-16
THC: CBG	0.94 (0.13, 1.76)	0.0224	LogLik.	-28.0586
CBG: CBD	0.39 (0.11, 0.67)	0.0067	S.D.	0.2350
			AIC	64.1172

Supplementary Table S6. E-Values of Spatiotemporal Regressions of Dataset Omitting Nevada

Parameter	R.R. (C.I.)	E-Values
CANNABIS		
Additive		
ASD ~ Cigarettes + Cannabis + Bng.Alc + Analgesics + Cocaine + Median.Income		
Cannabis	5.00 (2.10, 11.9)	9.48, 3.62
Interactive		
*ASD ~ Cigarettes * Cannabis + Bng.Alc + Analgesics + Cocaine + Median.Income*		
Cannabis	3.52 (1.40, 8.80)	6.49, 2.16
CANNABINOIDS		
Additive		
ASD ~ Cigarettes + THC + CBG + CBD + Bng.Alc + Analgesics + Cocaine + Median.Income		
CBD	1.46 (1.16, 1.84)	2.28, 1.60
Interactive x 3		
*ASD ~ Cigarettes * THC * CBG * CBD + Bng.Alc + Analgesics + Cocaine + Median.Income*		
THC: CBG	38.79 (1.69, 890.56)	77.08, 2.77
CBG: CBD	4.52 (1.52, 13.43)	8.51, 2.41

Supplementary Table S7. Spatiotemporal Regressions of Non-Overlapping Dataset

Parameters			Model	
**Parameter**	**Estimate (C.I.)**	**P-Value**	**Metric**	**Value**
CANNABIS			Psi	0.8047
Additive			Psi P-Value	6.83E-44
ASD ~ Cigarettes + Cannabis + Bng.Alc + Analgesics + Cocaine + Median.Income			LogLik.	-75.2964
Cannabis	1.45 (0.84, 2.06)	2.90E-06	S.D.	0.5152
Income	-1.43 (-2.65, -0.21)	0.0216	AIC	158.5927
Interactive			Psi	0.8047
*ASD ~ Cigarettes * Cannabis + Bng.Alc + Analgesics + Cocaine + Median.Income*			Psi P-Value	6.83E-44
Cannabis	1.45 (0.84, 2.06)	2.90E-06	LogLik.	-75.2964
Income	-1.43 (-2.65, -0.21)	0.0216	S.D.	0.5152
			AIC	158.5927
CANNABINOIDS			Psi	0.7726
Additive			Psi P-Value	9.54E-30
ASD ~ Cigarettes + THC + CBG + CBD + Bng.Alc + Analgesics + Cocaine + Median.Income			LogLik.	-79.6403
THC	1.37 (0.62, 2.11)	3.48E-04	S.D.	0.5559
Income	-1.39 (-2.77, -0.01)	0.0485	AIC	167.2806
Interactive x 3			Psi	0.8275
*ASD ~ Cigarettes * THC * CBG * CBD + Bng.Alc + Analgesics + Cocaine + Median.Income*			Psi P-Value	2.27E-66
THC : CBD	1.23 (0.47, 1.99)	0.0016	LogLik.	-81.4176
			S.D.	0.5454
			AIC	168.8352

Supplementary Table S8. Spatiotemporal Regressions of Temporally Lagged Dataset

Parameters			Model	
**Parameter**	**Estimate (C.I.)**	**P-Value**	**Metric**	**Value**
CANNABIS			Psi	0.9690
Additive			Psi P-Value	<2.0E-16
ASD ~ Cigarettes + Cannabis + Bng.Alc + Analgesics + Cocaine + Median.Income			LogLik.	-34.5585
Cannabis	0.52 (0.3, 0.73)	2.56E-06	S.D.	0.2391
			AIC	75.1170
Interactive			Psi	0.9704
*ASD ~ Cigarettes * Cannabis + Bng.Alc + Analgesics + Cocaine + Median.Income*			Psi P-Value	<2.0E-16
Cannabis	0.41 (0.18, 0.64)	4.23E-04	LogLik.	-31.4180
Cigarettes: Cannabis	-1.05 (-1.86, -0.24)	0.0114833	S.D.	0.2358
			AIC	70.8360
CANNABINOIDS			Psi	0.9694
Additive			Psi P-Value	<2.0E-16
ASD ~ Cigarettes + THC + CBG + CBD + Bng.Alc + Analgesics + Cocaine + Median.Income			LogLik.	-35.9896
THC	0.44 (0.08, 0.81)	0.017984	S.D.	0.2402
CBD	0.17 (0.09, 0.25)	2.16E-05	AIC	81.9791
CBG	-0.39 (-0.74, -0.04)	0.028117		
Interactive x 3			Psi	0.9694
*ASD ~ Cigarettes * THC * CBG * CBD + Bng.Alc + Analgesics + Cocaine + Median.Income*			Psi P-Value	<2.0E-16
THC	0.44 (0.08, 0.81)	0.017984	LogLik.	-35.9896
CBD	0.17 (0.09, 0.25)	2.16E-05	S.D.	0.2402
CBG	-0.39 (-0.74, -0.04)	0.028117	AIC	81.9791

SUPPLEMENTARY FIGURES S1-S13

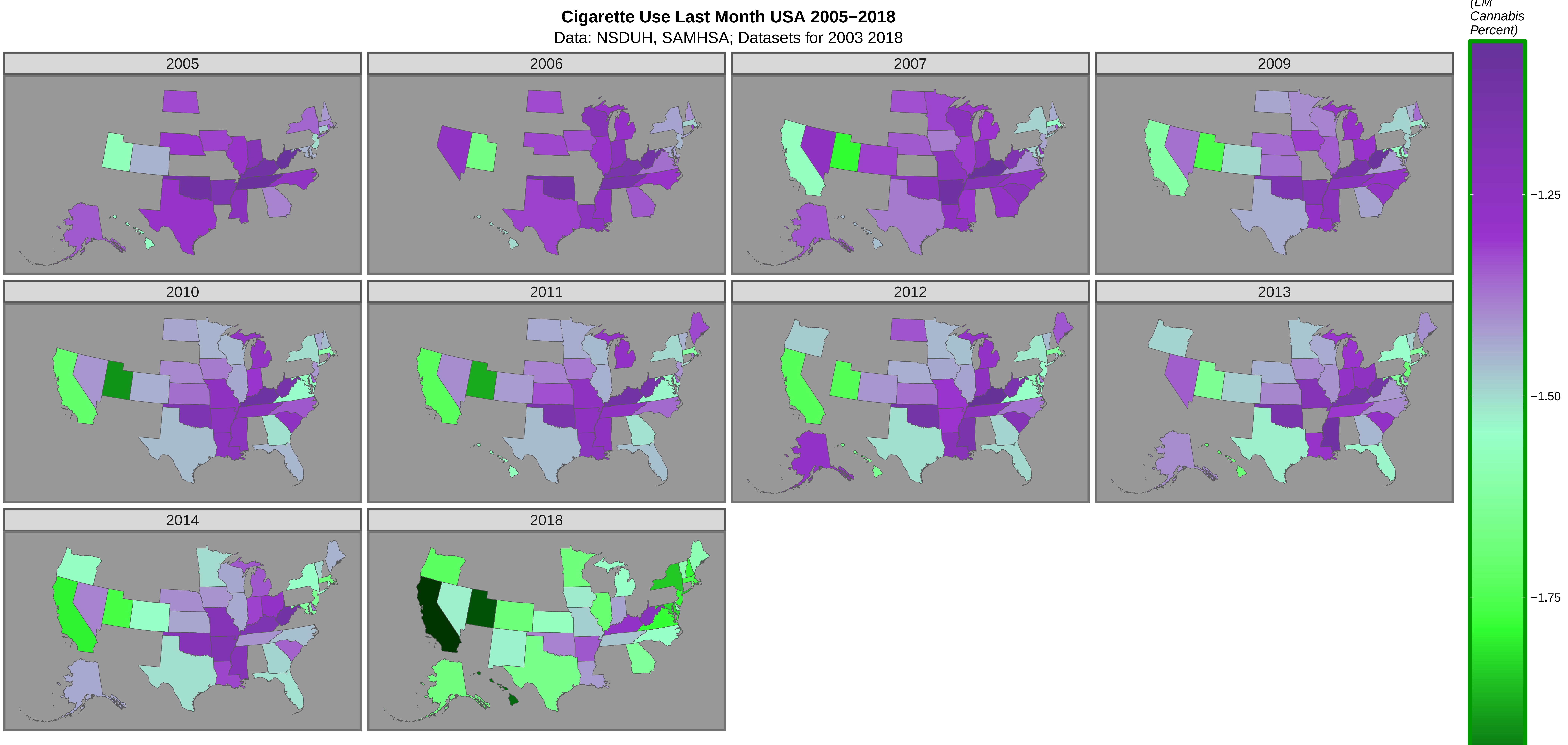
Cigarette Use Last Month USA 2005−2018
Data: NSDUH, SAMHSA; Datasets for 2003 2018
2005
2006
2007
2009
2010
2011
2012
2013
2014
2018
(LM
Cannabis
Percent)
−1.25
−1.50
−1.75

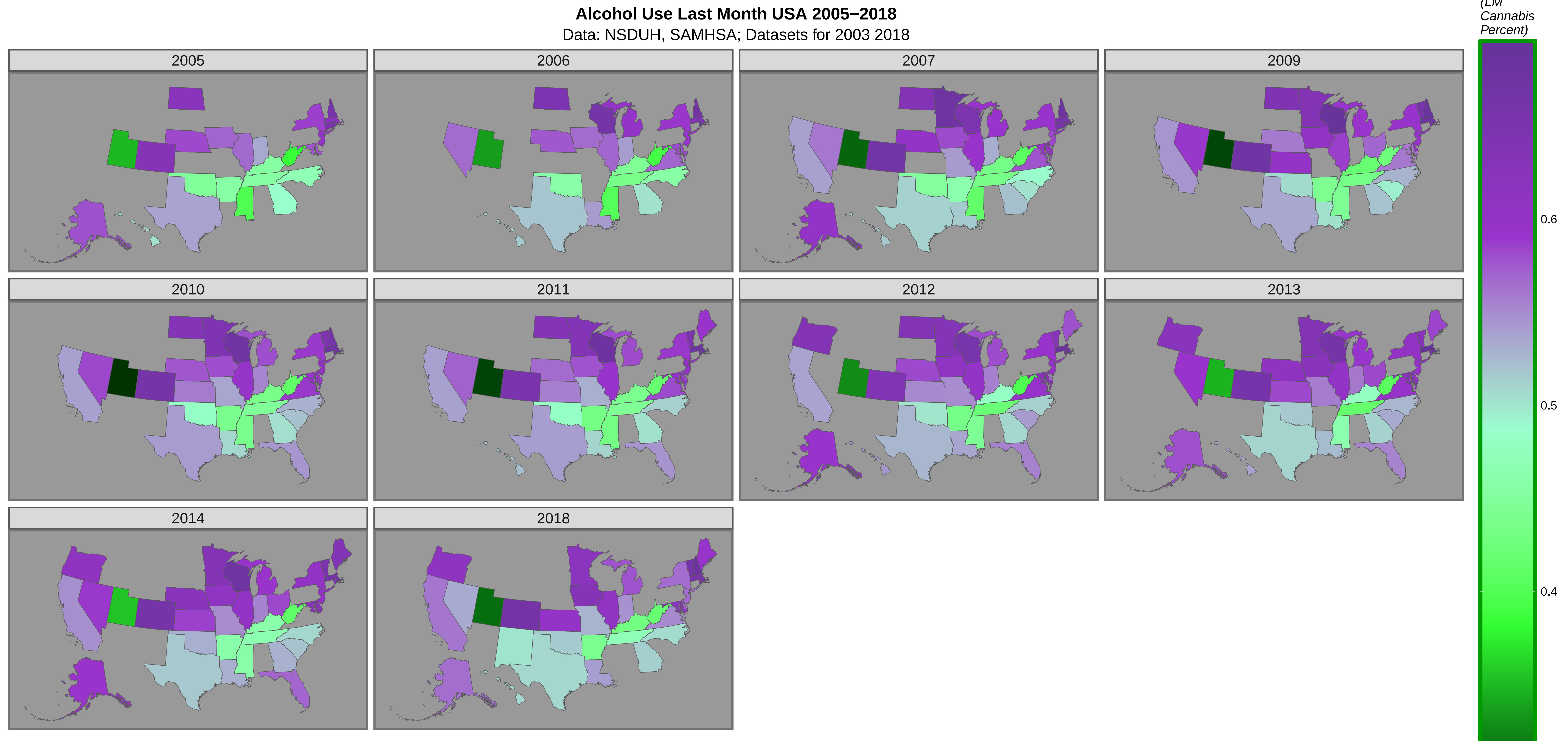
Alcohol Use Last Month USA 2005−2018
Data: NSDUH, SAMHSA; Datasets for 2003 2018
2005
2006
2007
2009
2010
2011
2012
2013
2014
2018
(LM
Cannabis
Percent)
0.6
0.5
0.4

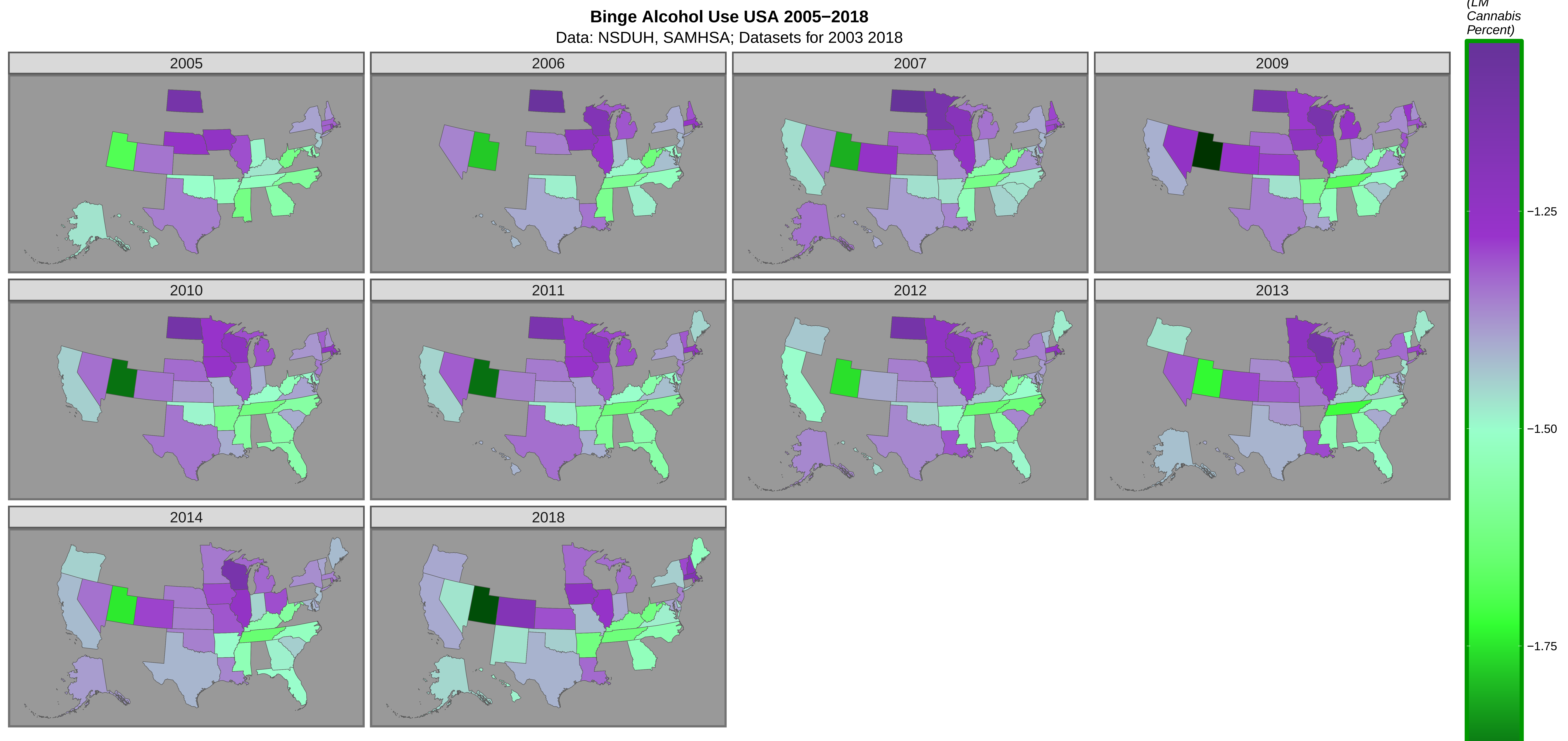
Binge Alcohol Use USA 2005−2018
Data: NSDUH, SAMHSA; Datasets for 2003 2018
2005
2006
2007
2009
2010
2011
2012
2013
2014
2018
(LM
Cannabis
Percent)
−1.25
−1.50
−1.75

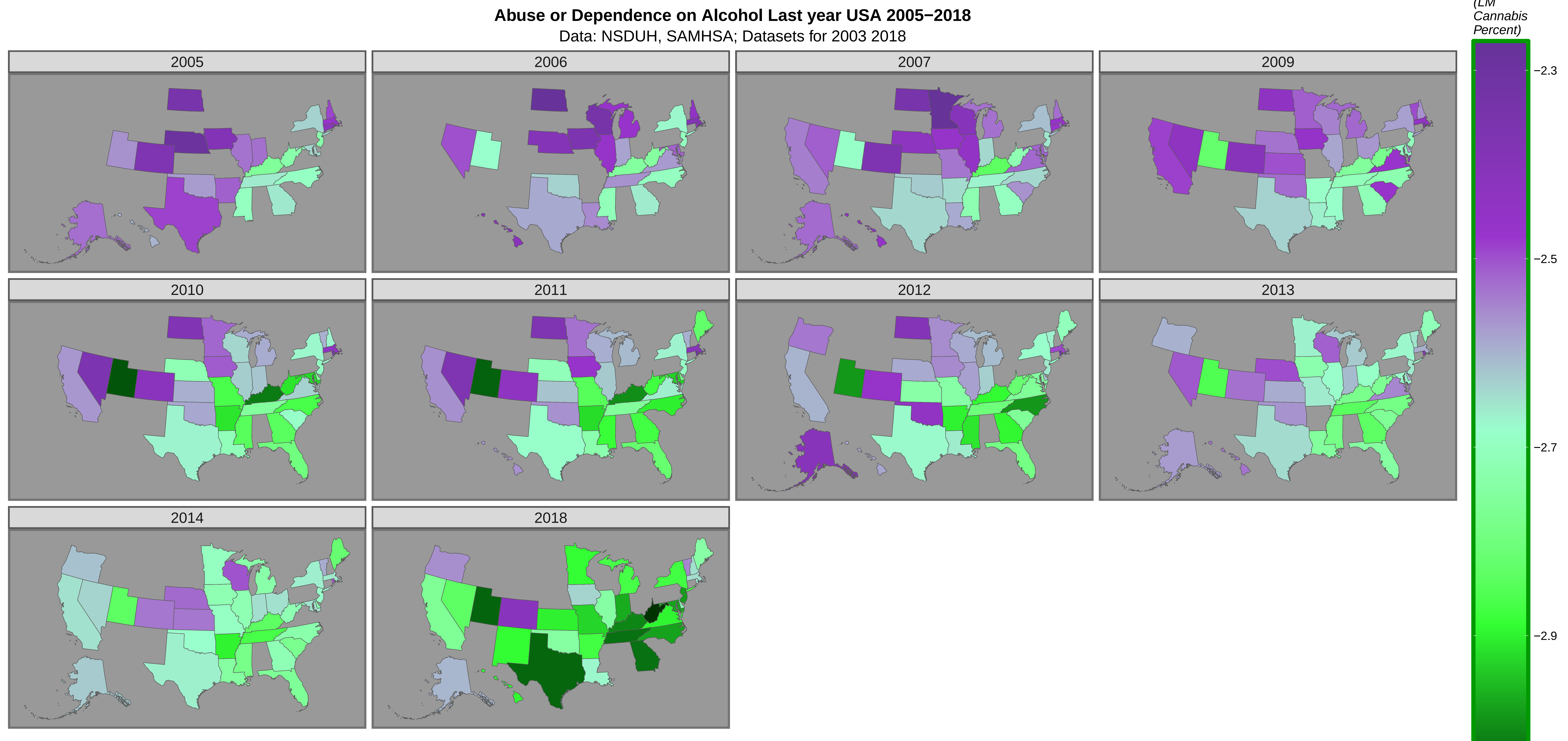
Abuse or Dependence on Alcohol Last year USA 2005−2018
Data: NSDUH, SAMHSA; Datasets for 2003 2018
2005
2006
2007
2009
2010
2011
2012
2013
2014
2018
Cannabis
Percent)
−2.3
−2.5
−2.7
−2.9

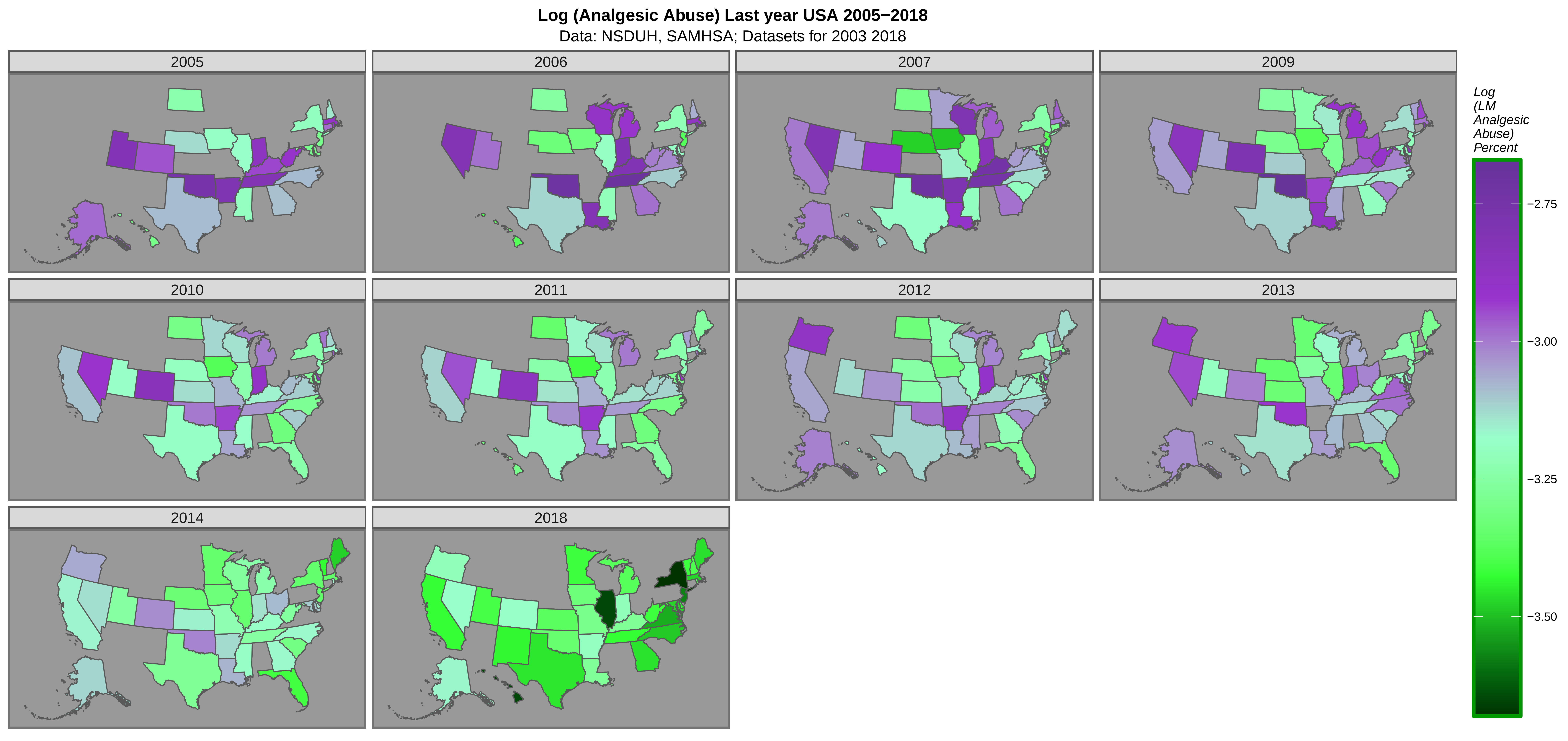

Log (Analgesic Abuse) Last year USA 2005−2018
Data: NSDUH, SAMHSA; Datasets for 2003 2018
2005
2006
2007
2009
2010
2011
2012
2013
2014
2018
Log (LM Analgesic Abuse) Percent
−2.75
−3.00
−3.25
−3.50

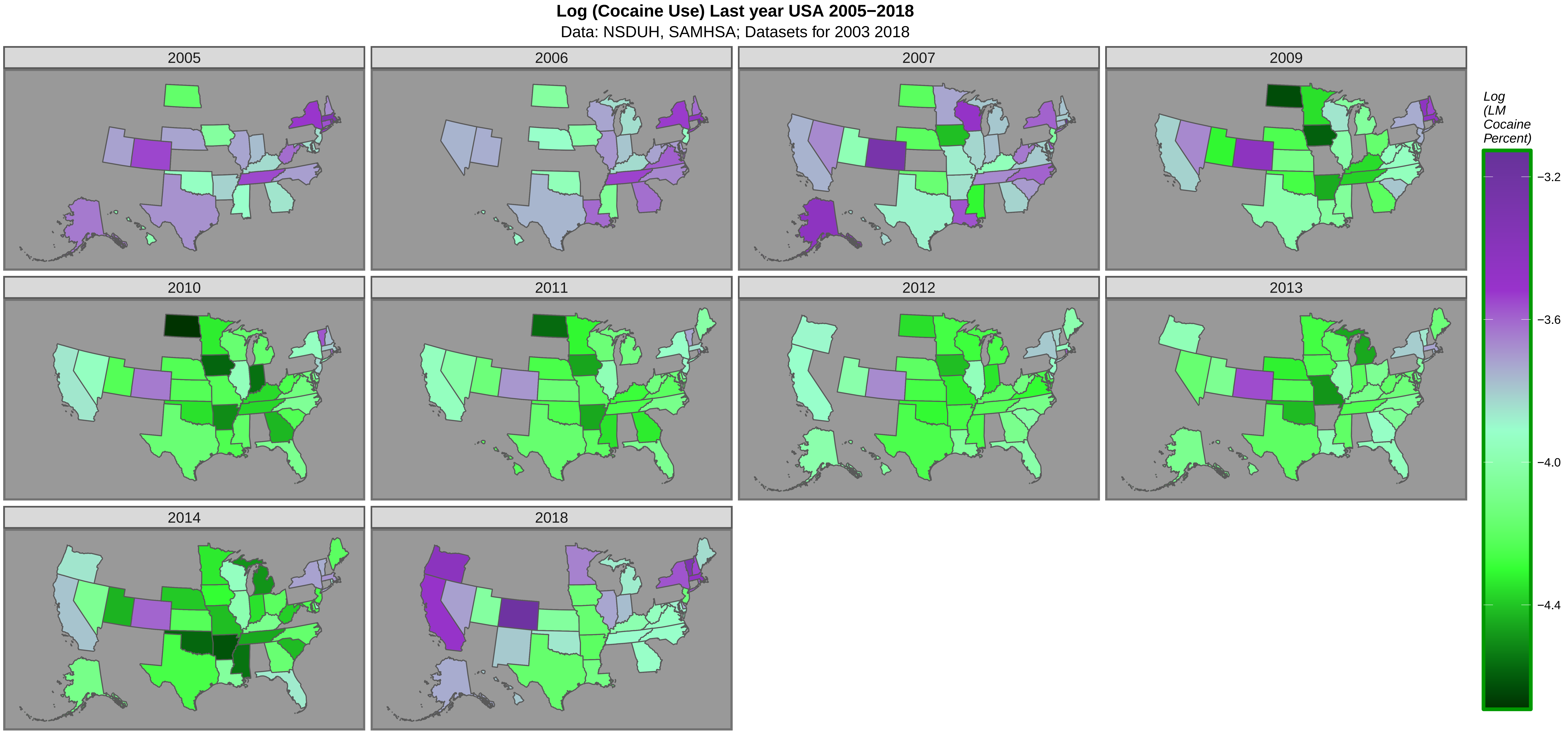
Log (Cocaine Use) Last year USA 2005−2018
Data: NSDUH, SAMHSA; Datasets for 2003 2018
2005
2006
2007
2009
2010
2011
2012
2013
2014
2018
Log (LM Cocaine Percent)
−3.2
−3.6
−4.0
−4.4

ASD−Secundum Rate by Monthly Tobacco Use by USA State
Bivariate Choropleth Colorplane Map

NBDPN of CDC Atlanta Georgia, to 2016−2020 Aggregated Data
and NSDUH SAMHSA DHHS USA to 2018

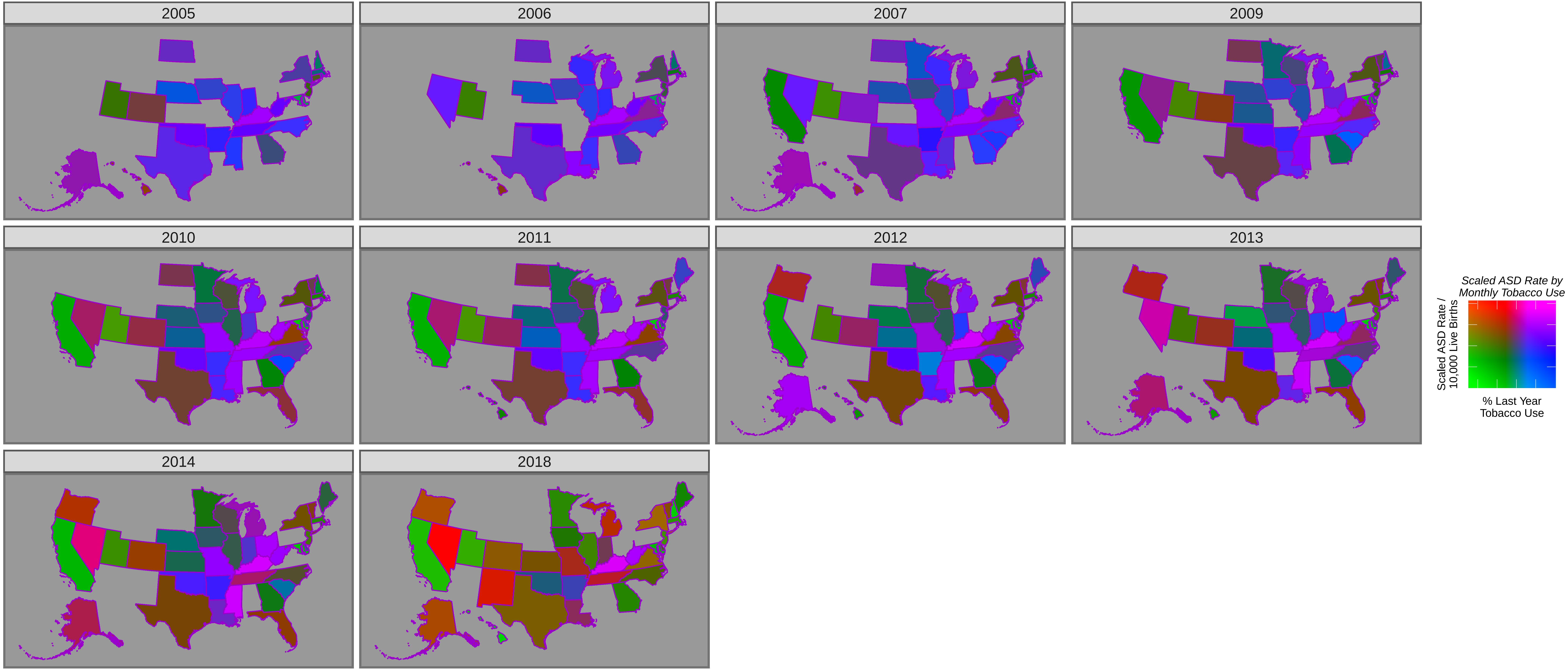

ASD−Secundum Rate by Annual Alcohol Dependence by USA State
Bivariate Choropleth Colorplane Map

NBDPN of CDC Atlanta Georgia, to 2016−2020 Aggregated Data
and NSDUH SAMHSA DHHS USA to 2018

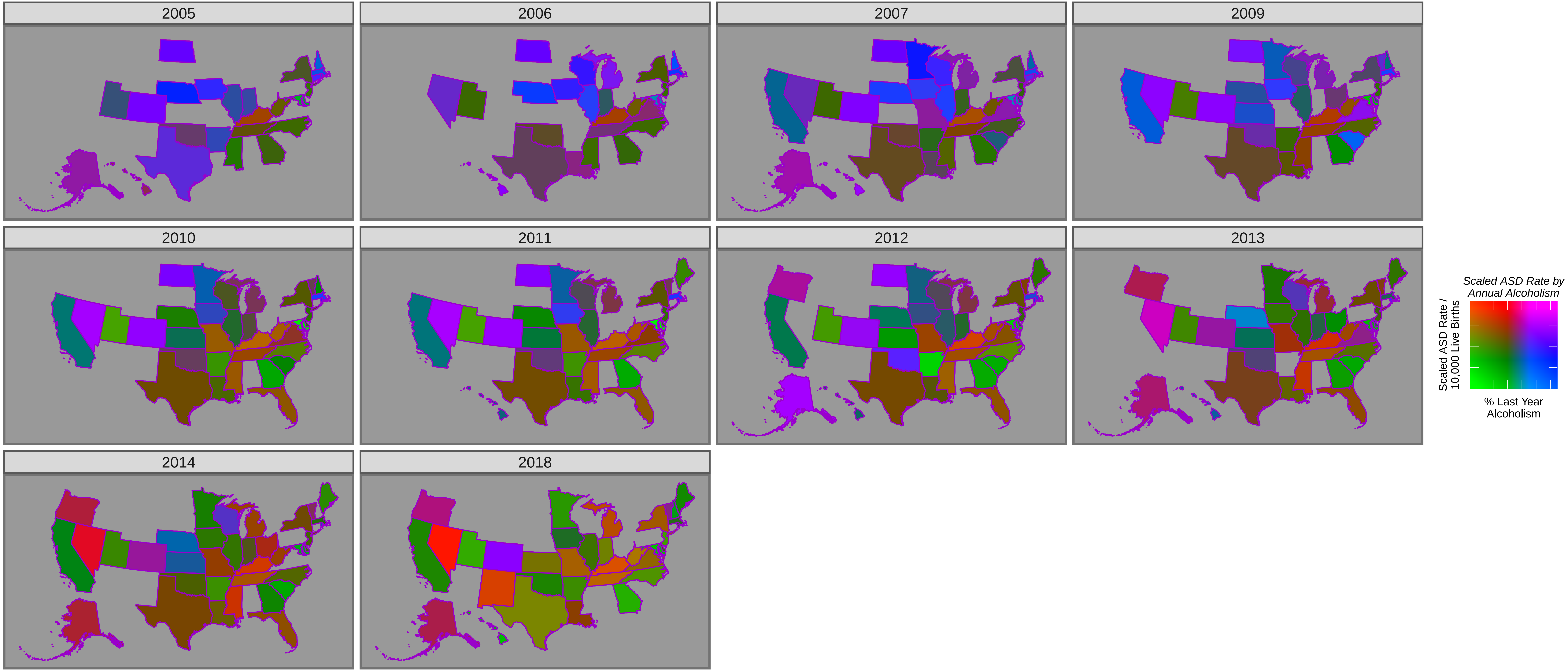

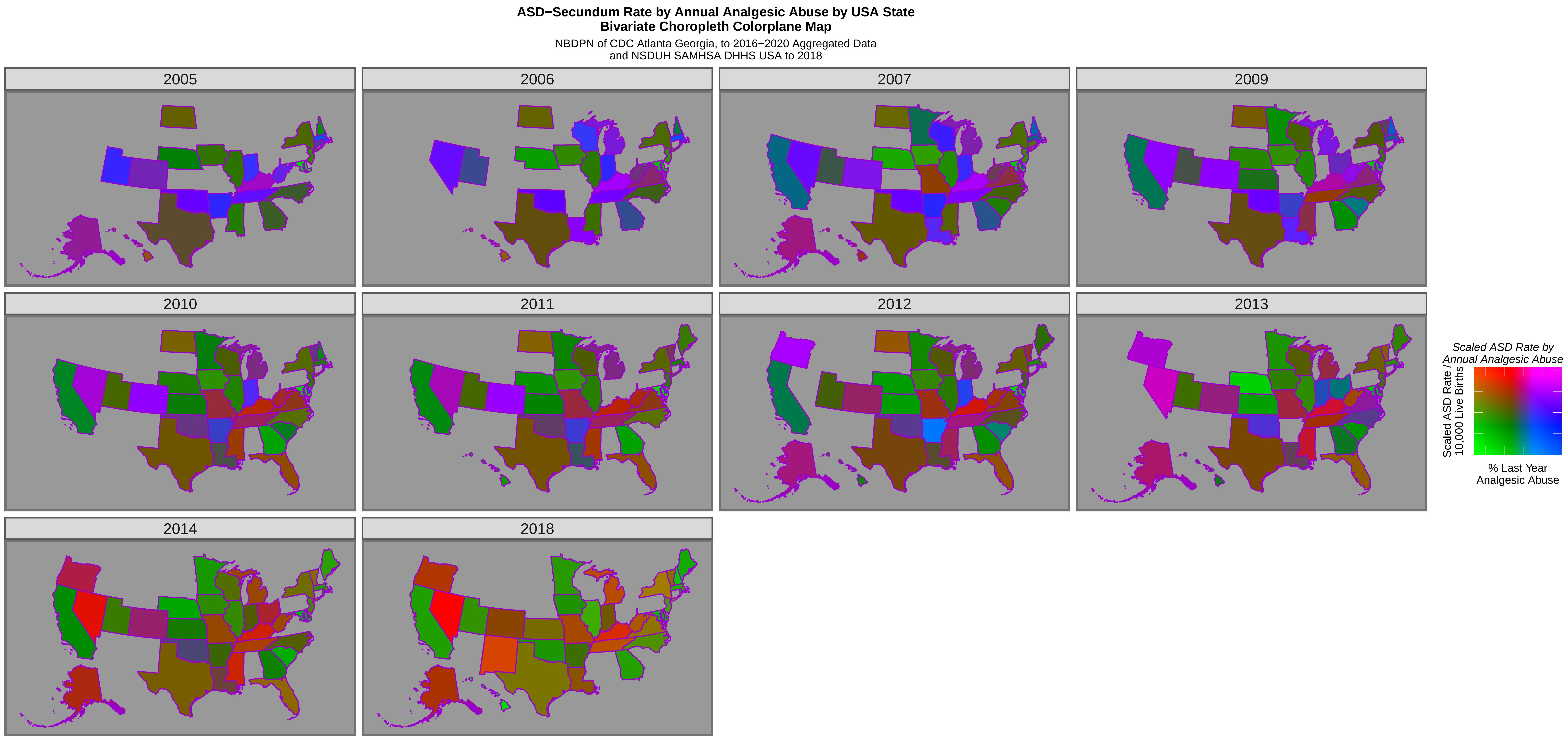

ASD−Secundum Rate by Annual Analgesic Abuse by USA State
Bivariate Choropleth Colorplane Map
NBDPN of CDC Atlanta Georgia, to 2016−2020 Aggregated Data
and NSDUH SAMHSA DHHS USA to 2018
2005
2006
2007
2009
2010
2011
2012
2013
2014
2018
Scaled ASD Rate by
Annual Analgesic Abuse
Scaled ASD Rate /
10,000 Live Births
% Last Year
Analgesic Abuse

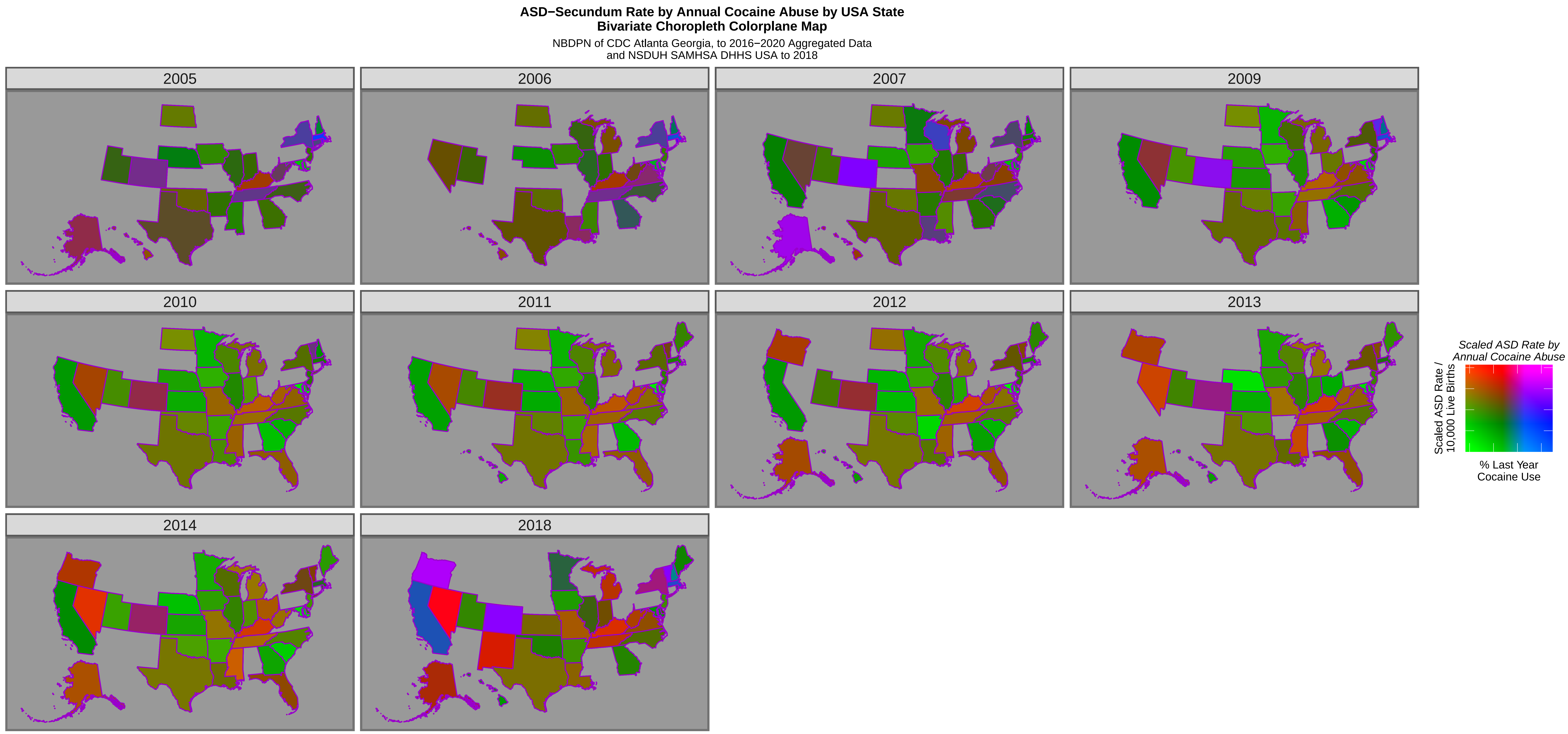

ASD−Secundum Rate by Annual Cocaine Abuse by USA State
Bivariate Choropleth Colorplane Map
NBDPN of CDC Atlanta Georgia, to 2016−2020 Aggregated Data
and NSDUH SAMHSA DHHS USA to 2018
2005
2006
2007
2009
2010
2011
2012
2013
2014
2018
Scaled ASD Rate by
Annual Cocaine Abuse
Scaled ASD Rate /
10,000 Live Births
% Last Year
Cocaine Use

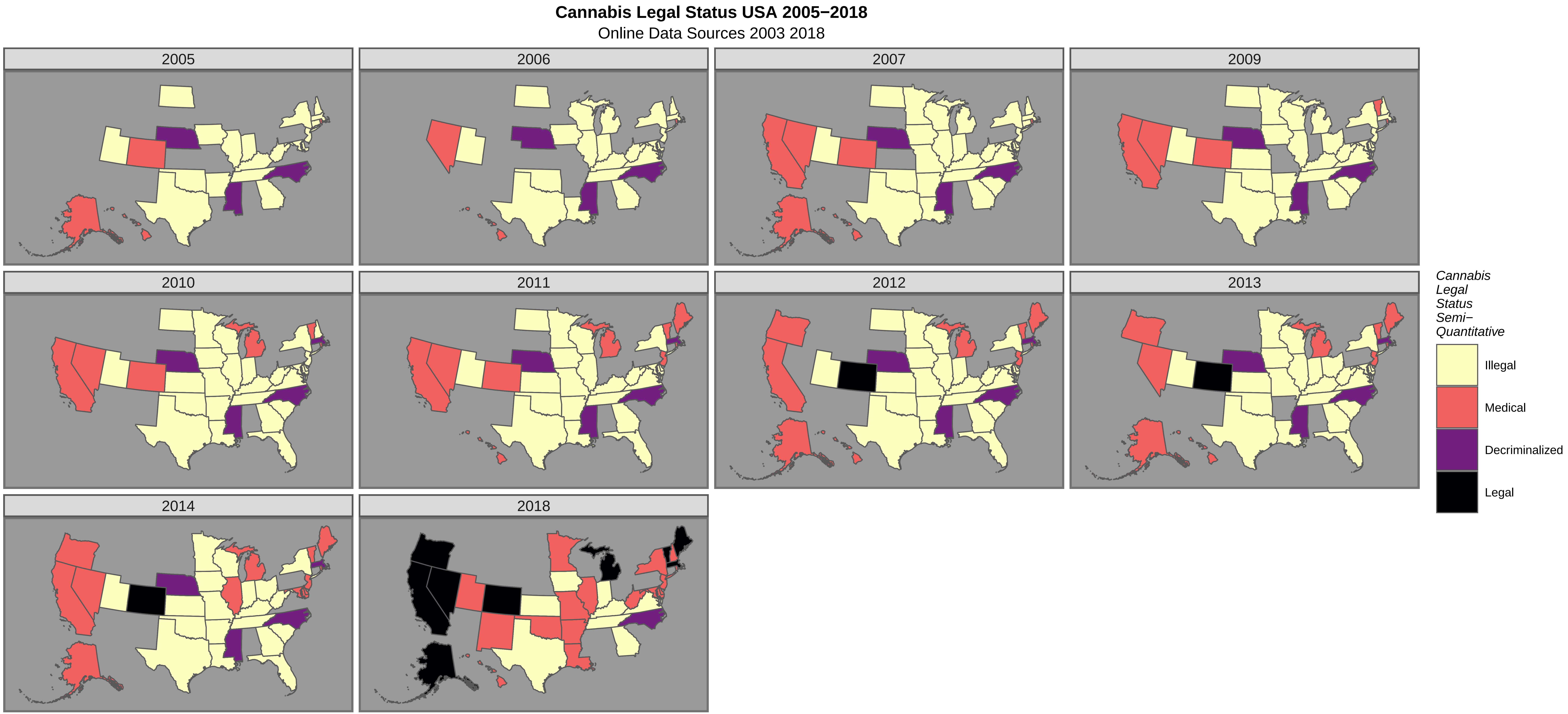
Cannabis Legal Status USA 2005−2018
Online Data Sources 2003 2018
2005
2006
2007
2009
2010
2011
2012
2013
2014
2018
Cannabis Legal Status Semi−Quantitative
Illegal
Medical
Decriminalized
Legal

Spatial LInks in Complete NBDPN ASD File

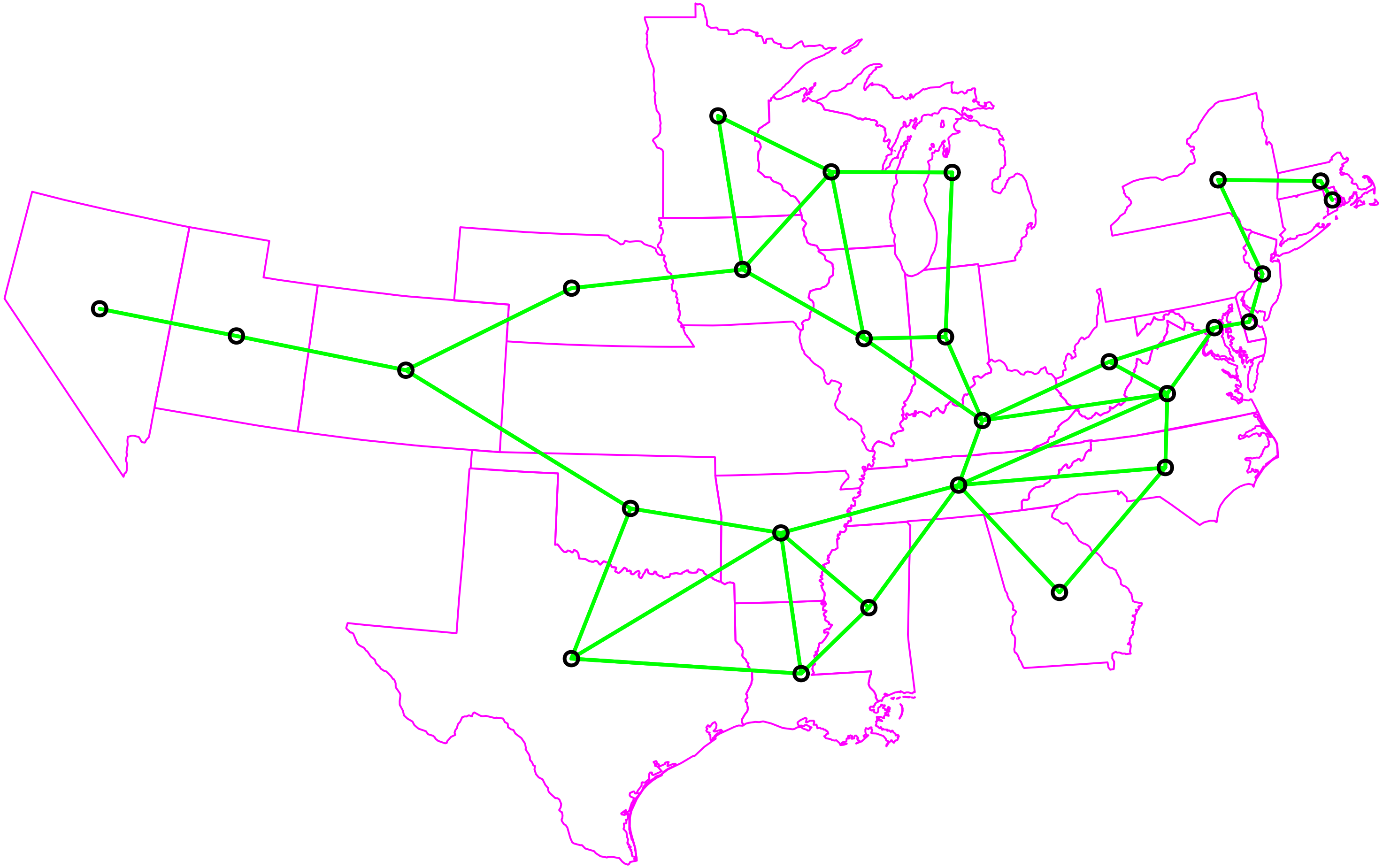

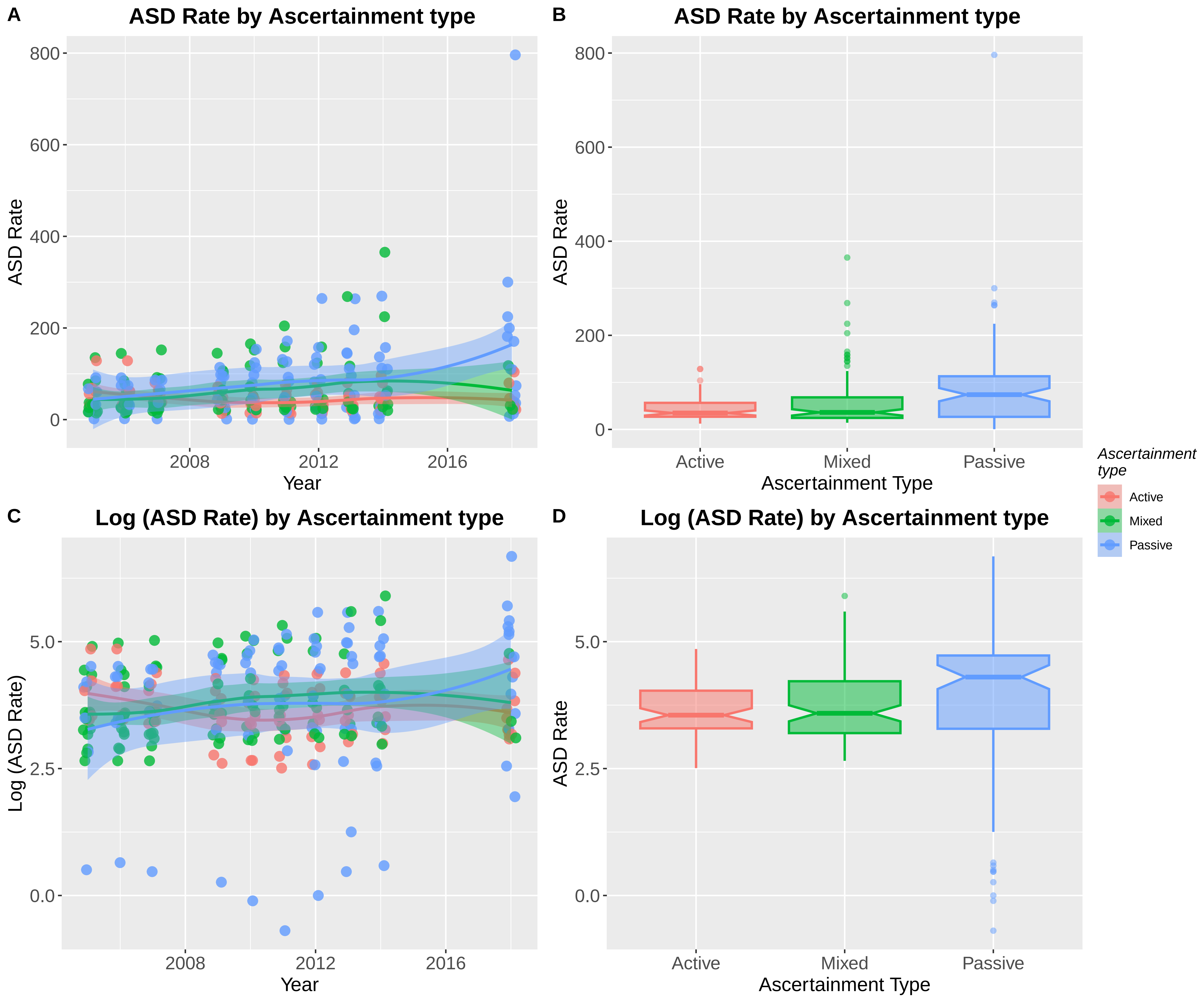
A
ASD Rate by Ascertainment type
ASD Rate
Year
B
ASD Rate by Ascertainment type
ASD Rate
Active
Mixed
Passive
Ascertainment Type
Ascertainment type
Active
Mixed
Passive
C
Log (ASD Rate) by Ascertainment type
Log (ASD Rate)
Year
D
Log (ASD Rate) by Ascertainment type
ASD Rate
Active
Mixed
Passive
Ascertainment Type
